# Selective biosynthesis of retinol in *S. cerevisiae*

**DOI:** 10.1186/s40643-022-00512-8

**Published:** 2022-03-12

**Authors:** Qiongyue Hu, Tanglei Zhang, Hongwei Yu, Lidan Ye

**Affiliations:** 1 Institute of Bioengineering, College of Chemical and Biological Engineering, Zhejiang University, Hangzhou, 310027 China; 2 Hangzhou Global Scientific and Technological Innovation Center, Zhejiang University, Hangzhou, 311200 China

**Keywords:** Vitamin A, Retinol, Biosynthesis, Metabolic engineering, Retinal reductase

## Abstract

The vitamin A component retinol has become an increasingly sought-after cosmetic ingredient. In previous efforts for microbial biosynthesis of vitamin A, a mixture of retinoids was produced. In order to efficiently produce retinol at high purity, the precursor and NADPH supply was first enhanced to improve retinoids accumulation in the *S. cerevisiae* strain constructed from a β-carotene producer by introducing β-carotene 15,15ʹ-dioxygenase, following by screening of heterologous and endogenous oxidoreductases for retinal reduction. Env9 was found as an endogenous retinal reductase and its activity was verified in vitro. By co-expressing *Env9* with the *E. coli ybbO*, as much as 443.43 mg/L of retinol was produced at 98.76% purity in bi-phasic shake-flask culture when the antioxidant butylated hydroxytoluene was added to prevent retinoids degradation. The retinol titer reached 2479.34 mg/L in fed-batch fermentation. The success in selective biosynthesis of retinol would lay a solid foundation for its biotechnological production.

**Graphical Abstract:**

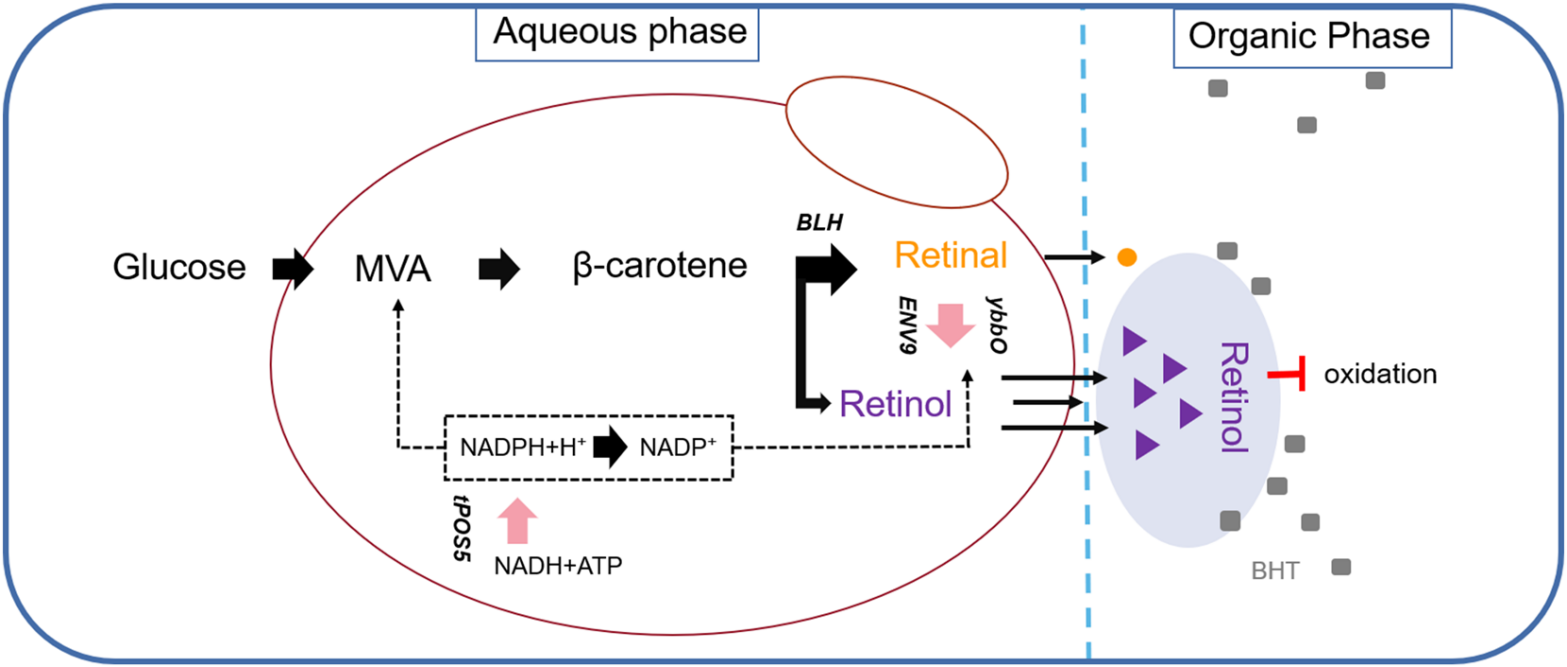

**Supplementary Information:**

The online version contains supplementary material available at 10.1186/s40643-022-00512-8.

## Introduction

As an essential vitamin for human body, vitamin A (retinoids) plays important roles in maintaining visual function, regulating cell differentiation and apoptosis, stabilizing epithelial cell morphology and function, development of B and T helper lymphocytes, and other physiological activities (D'Ambrosio et al. 2011; Olson and Mello 2010). Currently, vitamin A, including retinol and its active derivatives retinal and retinoic acid, is facing a growing market (Srinivasan and Buys 2019). Among the three forms, retinol has been used in cosmeceuticals as a main anti-aging ingredient ever since it was shown to have similar anti-wrinkle effects as retinoic acid but with fewer adverse effects (Rong et al. 2016). In addition, retinol has been utilized in manufacturing food products, pharmaceuticals, nutraceuticals and animal feed additives due to its anti-infective, anticancer and antioxidant functions (Hong et al. 2015).

**Fig. 1:**
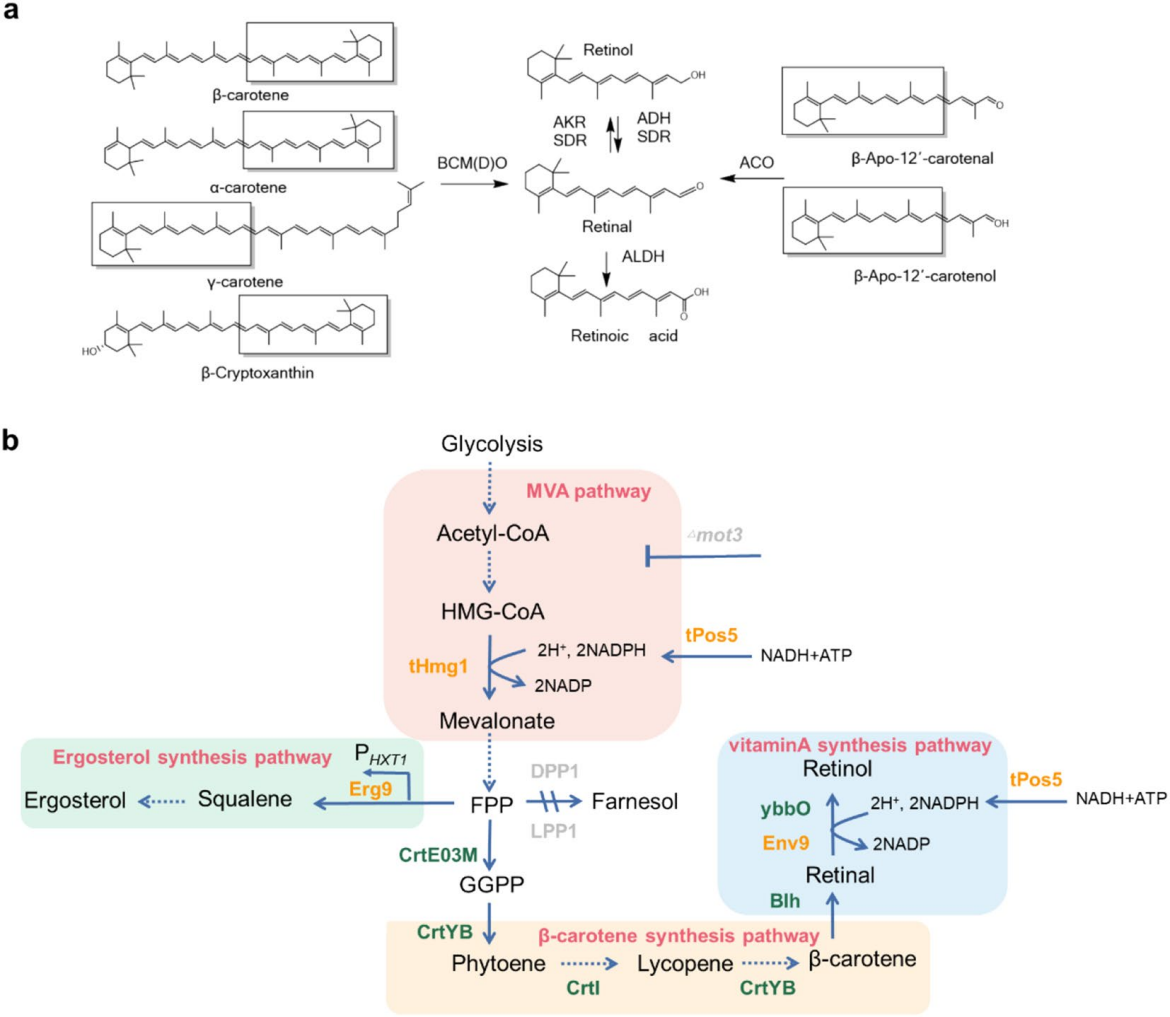
Vitamin A biosynthesis. **a** Biosynthesis of retinal and its conversion to other retinoids. BCDO, β-carotene 15,15ʹ-dioxygenase; BCMO, β-carotene 15,15′-monooxygenase; ACO, apo-carotenoid 15,15′-oxygenase; ALDH, aldehyde dehydrogenase; SDR, short-chain dehydrogenase/reductase; AKR, aldo-keto reductase; ADH, alcohol dehydrogenase. **b** Vitamin A biosynthetic pathway in *S. cerevisiae*. The biosynthetic pathway starts from the endogenous mevalonic acid (MVA) pathway, extended by a β-carotene synthetic pathway module consisting of *CrtE03M*, *CrtYB* and *CrtI* from *Xanthophyllomyces dendrorhous* and the vitamin A formation module containing the *Halobacterium sp.* β-carotene dioxygenase gene *BLH* and the retinal reductase genes (*ybbO* from *E. coli* and *ENV9* from *S. cerevisiae*). The major competing branches for the key precursor farnesyl pyrophosphate (FPP) include the ergosterol synthetic pathway and the phosphatase-catalyzed formation of farnesol. Diacylglycerol pyrophosphate phosphatase gene *DPP1* and lipid phosphate phosphatase gene *LPP1* were knocked out and used as integration sites. The exogenous enzymes introduced are shown in green, the endogenous enzymes are depicted in orange and those deleted are depicted in gray. HMG-CoA, 3-hydroxy-3-methyl-glutaryl-CoA; FPP, farnesyl pyrophosphate; GGPP, geranylgeranyl diphosphate; tHmg1, truncated 3-hydroxy-3-methyl-glutaryl reductase; CrtE, geranylgeranyl diphosphate synthase; CrtI, phytoene desaturase; CrtYB, phytoene synthase/ lycopene cyclase; Blh, bacterirhodopsin-related-protein-like homolog protein (later identified as β-carotene dioxygenase); ybbO, aldehyde reductase; tPos5, truncated NADH kinase

In nature, vitamin A is a degradation product of carotenoids known as provitamin A (Chapman 2012; Kim and Oh 2010; Scherzinger et al. 2006). These carotenoids are naturally synthesized in photosynthetic organisms, and can release at least one molecule of retinal under catalysis of a proper carotenoid cleavage dioxygenase (Fig. 1a). In particular, the central double bond cleavage reaction mediated by β-carotene 15,15ʹ-mono(di)oxygenases could produce two molecules of retinal from one molecule of β-carotene. Retinal can then be converted to retinol and retinoic acid via bio-reduction or oxidation reactions, respectively. Heterologous biosynthesis of vitamin A was first reported in a β-carotene-producing *E. coli* by introducing the *BLH* gene encoding β-carotene dioxygenase from the archaeon *Halobacterium* sp. NPC-1, leading to production of 136 mg/L retinoids, which was a mixture of retinol, retinal and retinyl acetate (Jang et al. 2011; Peck et al. 2001; Sabehi et al. 2005). More recently, retinoids production from xylose using an engineered *S. cerevisiae* strain was reported, and retinoids were produced with a retinal/retinol ratio of 1.67 (Sun et al. 2019). In all these studies, a mixture of retinoids was produced even though no retinal reductase/oxidase was introduced. In *E. coli*, the aldehyde reductase ybbO had been found to increase the retinol proportion in retinoids from 38 to 53% when overexpressed (Jang et al. 2015). However, the enzyme(s) responsible for retinol formation during vitamin A biosynthesis in *S. cerevisiae* have not yet been identified. Mining and overexpression of endogenous and exogenous enzymes with retinal reductase activity is expected to improve the retinol proportion in the yeast-synthesized vitamin A, and would possibly lead to selective biosynthesis of retinol with a higher market value.

To improve the titer of retinol in *S. cerevisiae*, strategies that have been proven efficient in boosting carotenoids biosynthesis could be adopted, such as strengthening the precursor supply by overexpression of geranylgeranyl pyrophosphate (GGPP) synthase (Ignea et al. 2015), repression of the competing squalene synthesis pathway (Zha et al. 2020), and deletion of repressor genes such as *ROX1, MOT3* and a distant genetic locus, *YPL062W* (Chen et al. 2016; Hong et al. 2019). Considering the NADPH requirement of the 3-hydroxy-3-methyl-glutaryl reductase(Hmg1)-catalyzed reaction, elevation of NADPH supply is shown as another powerful approach to elevate the flux of the mevalonic acid (MVA) pathway, contributing to the production of terpenoids (Hong et al. 2019; Zhao et al. 2013). In the oxidoreductase-mediated retinal conversion to retinol, sufficient supply of redox potential would also be a necessity.

As an outstanding antioxidant, retinol is easily oxidized or isomerized when exposed to light, heat and oxygen due to the continuous conjugated double bond chains in their structures (Sauvant et al. 2012). In an experiment on the stability of vitamin A against UVA and UVB rays, the antioxidant butylated hydroxytoluene (BHT) exhibited excellent protective function (Carlotti et al. 2002). Compared with solid lipid nanoparticles, cyclodextrin inclusion compound and polymer encapsulation, addition of BHT was considered as a simple, cheap and safe method to prevent the degradation of retinol (Loveday and Singh 2008). Therefore, addition of BHT during retinol fermentation may prevent its degradation and contribute to its accumulation.

In this study, we first constructed a retinoids-producing yeast by introducing *BLH* into the previously constructed β-carotene producer Ycarot-02 (Zhou et al. 2017). The production of retinoids was then improved by pathway optimization towards enhanced precursor supply and coenzyme pool expansion. Subsequently, dehydrogenases from different sources were introduced into the vitamin A-producing yeast to compare their capability of retinal reduction, and endogenous retinal reductases were mined based on sequence alignment and experimental verification. The effect of BHT addition on the production of retinoids was then investigated. Finally, an engineered yeast strain exclusively producing retinol was obtained and examined for production capability in high-density fermentation.

## Results and discussion

### Construction of vitamin A synthesis pathway

**Fig. 2:**
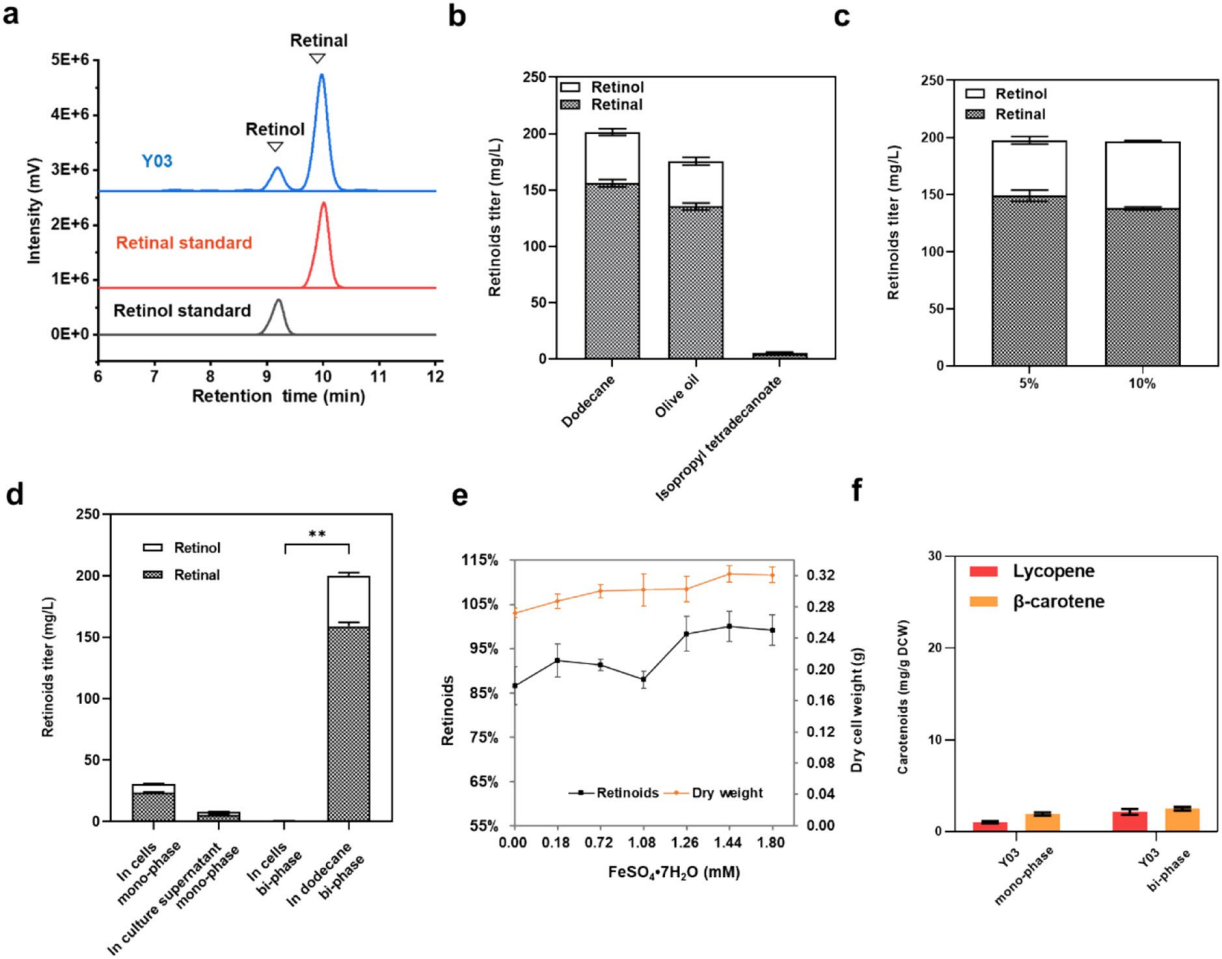
Retinoids biosynthesis in the engineered yeast strain Y03. **a** HPLC chromatograms of strain Y03, together with the retinal and retinol standards. **b** Effect of extractant type on bi-phasic fermentation of retinoids. **c** Effect of dodecane overlay volume on retinoids production. **d** Distribution of retinoids in mono- and bi-phasic fermentation. **e** Effect of Fe^2+^ addition on retinoids production. **f** Intracellular accumulation of carotenoids in mono- and bi-phasic fermentation. Statistical significance was evaluated using Student’s *t* test (*, *P* < 0.05; **, *P* < 0.01)

Based on the previously constructed *S. cerevisiae* strain Ycarot-02 (Zhou et al. 2017) harboring the β-carotene biosynthesis pathway, a complete vitamin A synthetic pathway was assembled by integration of the *BLH* gene from the uncultured marine bacterium MED66A03, generating strain Y03 (Fig. 1b). As expected, retinoids were detected in Y03 (Fig. 2a). In consistence with previous studies on microbial synthesis of retinoids (Jang et al 2015; Sun et al. 2019), retinol was detected in addition to retinal, indicating the presence of endogenous dehydrogenases with retinal reduction activity.

Organic solvents with high biocompatibility and low cost were used to extract hydrophobic compounds from the host in bi-phasic cultivation, which relieved the product toxicity to the cells, increased the accumulation of product and prevented its volatilization (Brennan et al. 2012; Hoshino et al. 2020). Since the type of the organic solvent influences the extraction efficiency, we compared the retinoids production in bi-phasic culture using 5% (v/v) dodecane, olive oil and isopropyl tetradecanoate as the extractant, respectively (Fig. 2b). Dodecane was found the most efficient, but increasing the volume ratio from 5% (v/v) to 10% (v/v) did not further improve retinoids production (Fig. 2c). In the bi-phasic culture with 5% (v/v) dodecane as the overlay, almost all retinoids (99.67%) were extracted to the upper layer, and the titer was 5.21 times (200.21 mg/L vs. 38.41 mg/L) higher than that in monophase culture (Fig. 2d). Surprisingly, addition of isopropyl tetradecanoate to the culture almost completely inhibited retinoids production by blocking β-carotene formation as judged by the cell pellet color and the absence of β-carotene peak in HPLC analysis (Additional file 1: Figure S1), although this organic solvent had been previously reported to improve linalool and lupeol production when added as the in situ extractant (Hoshino et al. 2020; Zhang et al. 2020). This result implied that isopropyl tetradecanoate may have a negative effect on the expression or activity of lycopene β-cyclase, but the mechanism remains to be explored.

Since Blh was reported to be a Fe^2+^-dependent protoporphyrin (non-heme iron) enzyme (Kim et al. 2009), the Fe^2+^ concentration in the medium was optimized. When the concentration of Fe^2+^ was 1.44 mM, the total titer of retinoids reached a peak and the cell growth was also slightly improved (Fig. 2e), possibly because Fe^2+^ as a cofactor in numerical biological processes promotes the reproduction, lipids biosynthesis and oxygen transport (Ramos-Alonso et al. 2020).

### Enhancement of precursor supply

**Fig. 3:**
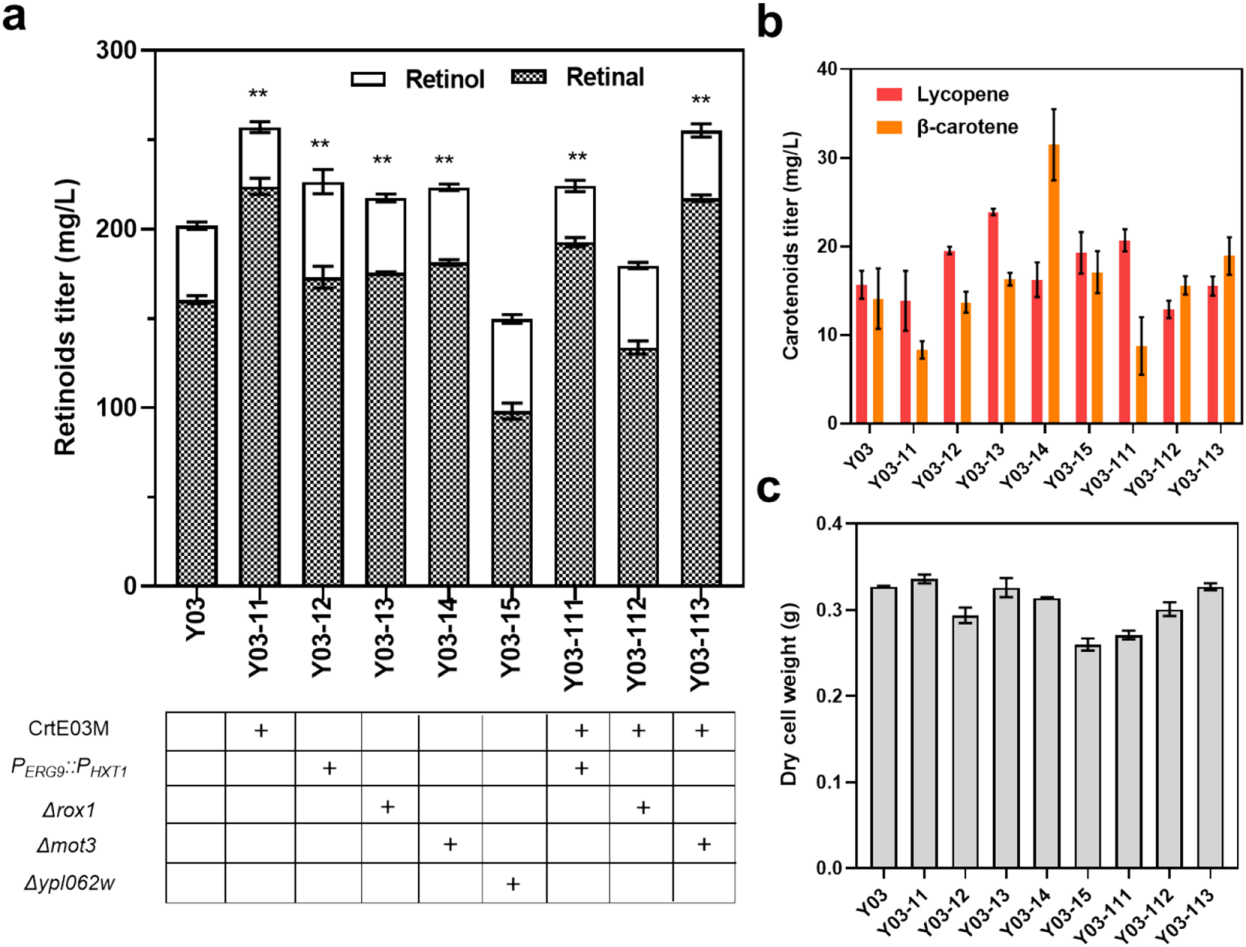
Effect of *CrtE03M* overexpression, *ERG9* downregulation, and deletion of *ROX1, MOT3* or *YPL062W* on retinoids titer (**a**), carotenoids titer (**b**), and dry cell weight (**c**). The error bars represent standard deviations calculated from triplicate experiments, and statistical significance of the different retinoids levels in comparison with the control was evaluated using Student’s *t* test (*, *P* < 0.05; **, *P* < 0.01)

Efforts to improve retinoids production by increasing the copy number of *BLH* and *CrtYB* failed (Additional file 1: Figure S2), suggesting that the rate-limiting step might lie in the endogenous precursor supply rather than the heterologous pathway modules. In accordance, carotenoids accumulated in cells of strain Y03 in either mono- or bi-phasic cultures were quite low (Fig. 2f), confirming that the precursor supply might be limiting. Therefore, we turned to strengthen the supply of the key precursor GGPP. In strain Ycarot-02 (Zhou et al. 2017), a positive mutant of CrtE (CrtE03M) from *Xanthophyllomyces dendrorhous* had been introduced to enhance the supply of GGPP. When another copy of *CrtE03M* was introduced, the titer of retinoids was further increased by 27.33% in Y03-11 (Fig. 3a), confirming the supply of GGPP as a limiting factor. Conditional downregulation of the competing squalene synthetic pathway was employed as an alternative approach to enhance GGPP supply. By replacing the native promotor of *ERG9* in Y03 with the glucose-induced promotor P_*HXT1*_ (Xie et al. 2015b), the retinoids titer was improved by 12.19%, but the cells grew very slowly and the biomass decreased by 10.09% (Fig. 3c) due to the important role of squalene-derived ergosterol in the development of cell membrane (Baadhe et al. 2013). Impaired cell growth was also observed in strain Y03-111 with both *CrtE03M* overexpression and *ERG9* downregulation, which resulted in lower retinoids titer than Y03-11, although its specific cellular production of retinoids was higher than that of Y03-11 (41.36 mg/g DCW vs. 38.26 mg/g DCW). Therefore, strain Y03-11 was used in further studies.

Deletion of *ROX1, MOT3* or *YPL062W* is another frequently used strategy to improve precursor supply for isoprenoids production. Rox1 and Mot3 are transcriptional repressors of the MVA pathway (Hu et al. 2020; Jorda and Puig 2020) while *YPL062W* deletion is assumed to help “trapping” the carbon from acetate accumulation toward acetyl-CoA (Chen et al. 2016). However, retinoids production dramatically decreased in the *Δypl062w* strain, whereas deletion of *ROX1* or *MOT3* showed little impact on retinoids production*.* Nevertheless, accumulation of β-carotene was obviously enhanced by *MOT3* knockout (Fig. 3b). In strain Y03-11, deletion of *MOT3* also did not affect retinoids synthesis. Therefore, *MOT3* was used as an integration site in later manipulations.

### Elevation of intracellular NADPH supply

As the well-recognized rate-limiting enzyme of the MVA pathway, truncated Hmg1 (tHmg1) had been overexpressed in the parent strain Ycarot-02 to promote isopentenyl pyrophosphate (IPP)/dimethylallyl pyrophosphate (DMAPP) supply for isoprenoids synthesis (Zhou et al. 2017). In the Hmg1-catalyzed reaction, NADPH is involved as a cofactor, the supply of which would influence the catalytic activity of Hmg1 and thus the MVA pathway flux.

**Fig. 4:**
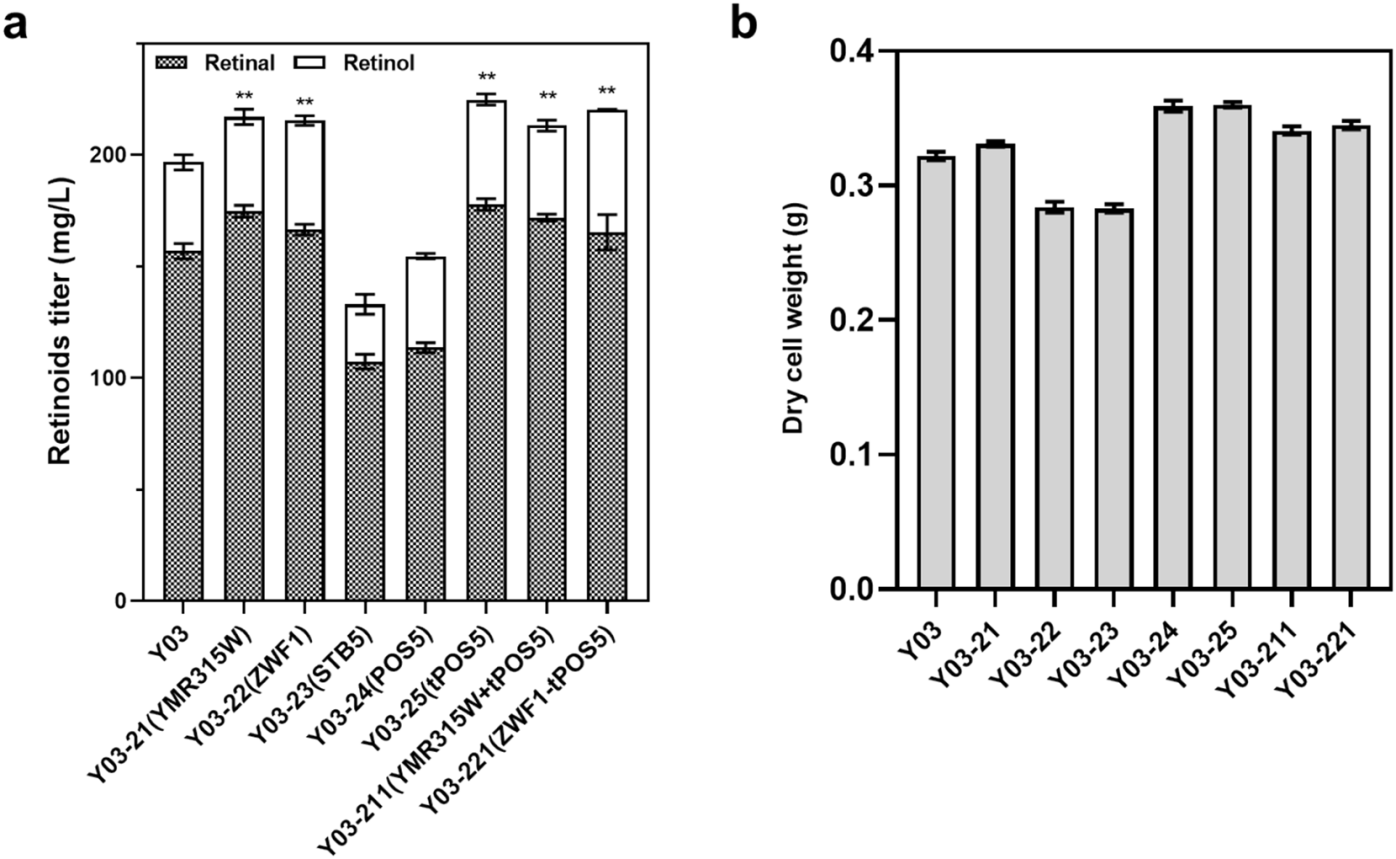
Effect of enhancing NADPH supply on retinoids production. **a** Retinoids titer and **b** dry cell weight of each strain are indicated. The error bars represent standard deviations calculated from triplicate experiments, and statistical significance of the different retinoids levels in comparison with the control was evaluated using Student’s *t* test (*, *P* < 0.05; **, *P* < 0.01)

The two NADP(H) oxidoreductases Zwf1 and YMR315W and their transcription factor Stb5, as well as the mitochondrial NADH kinase Pos5 were overexpressed in Y03, respectively, to elevate the intracellular supply of NADPH. As the first enzyme of the pentose phosphate pathway, the Zwf1-catalyzed reaction generates a majority of NADPH in the cytosol. YMR315W, whose promoter contains a putative Stb5 binding site, was also reported to contribute to NADPH production (Hector et al. 2009; Hong et al. 2019). When YMR315W or Zwf1 was overexpressed, slight increase in retinoids production was observed (Fig. 4a). However, Stb5 overexpression failed to improve retinoids biosynthesis.

Pos5 was reported to mediate the ATP-driven conversion of NADH into NADPH in the yeast mitochondria (Hou et al. 2009). To allow Pos5 working in the cytosol, the 17 aa mitochondrial signal peptide was excised, generating the truncated protein tPos5. Overexpression of tPos5 rather than the intact Pos5 in strain Y03 led to 14.32% higher retinoids production in Y03-25 together with 11.80% improvement in cell dry weight in the resulting strain Y03-25 (Fig. 4b). Overexpression of tPos5 increased the total titer of retinoids, not only in retinal production, but also in retinol production (39.81 mg/L vs. 47.01 mg/L), implying the involvement of NADPH in the conversion of retinal to retinol by the endogenous dehydrogenases. However, co-overexpression of tPos5 and Zwf1 or tPos5 and Ymr315w did not lead to further increase in retinoids production. Although the retinol proportion was improved (24.87% vs. 20.91%), the growth and retinoids titer of the resulting strain Y03-221 and Y03-211 were slightly lower than those of Y03-25. Meanwhile, the NADPH level and NADPH/NADH ratio both increased after overexpression of tPos5 (Table 1).

**Table 1:** Detection of intracellular NADPH and NADH

Strain	NADH (μmol/g DCW)	NADPH (μmol/g DCW)	NADPH/NADH ratio
Y03	0.87 ± 0.01	0.10 ± 0.004	0.11
Y03-25(*tPOS5*)	0.62 ± 0.02	0.21 ± 0.005	0.34

### Heterologous expression of retinal reductases

**Fig. 5:**
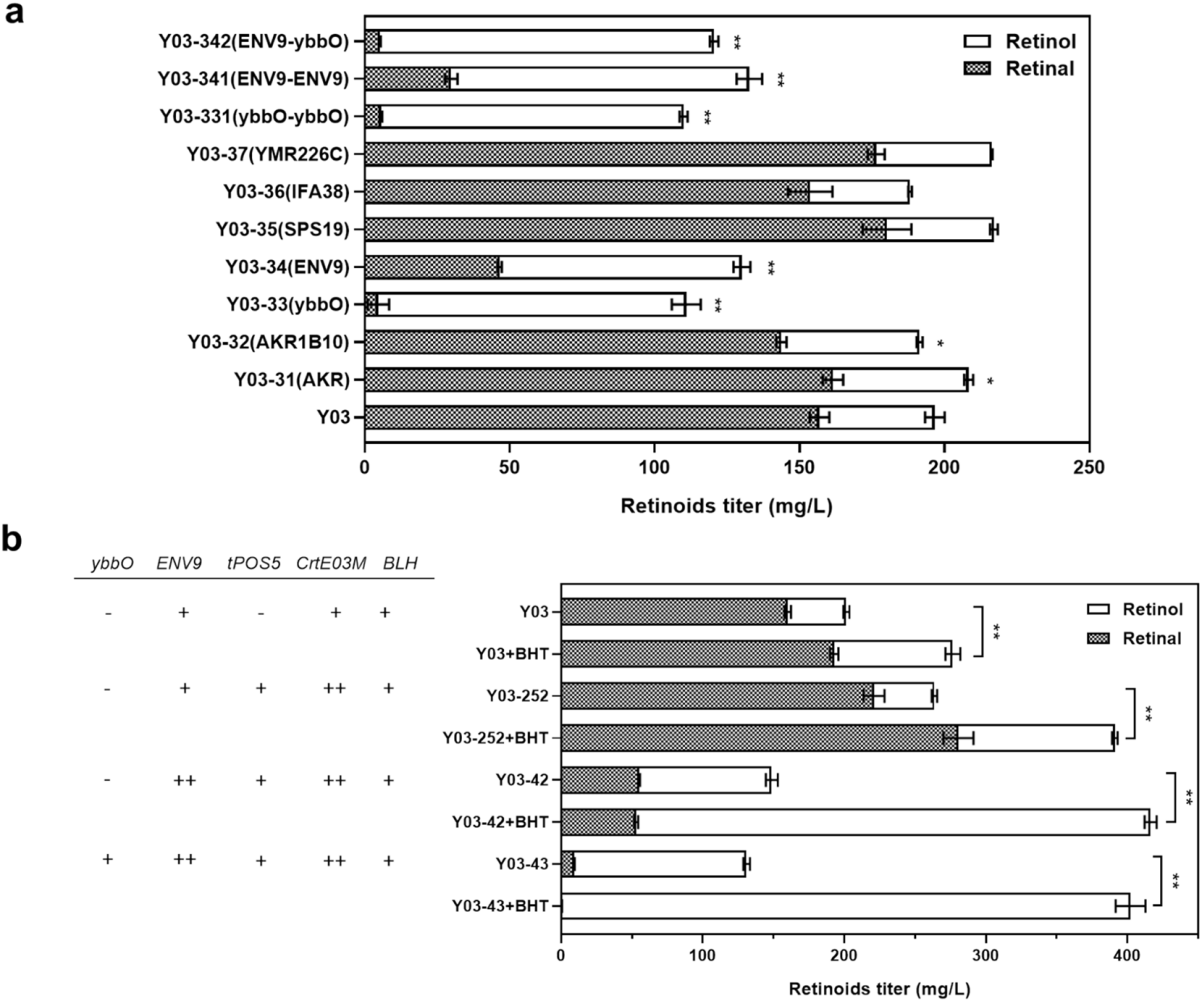
Enhancement of retinol production through mining and evaluation of endogenous and exogenous alcohol dehydrogenases and investigation of the role of antioxidant BHT. **a** Effect of overexpressing endogenous or exogenous dehydrogenases on retinol formation. **b** Retinoids production by the engineered yeast strains in the absence and presence of BHT. The error bars represent standard deviations calculated from triplicate experiments and statistical significance of the different retinol levels in comparison with the control was evaluated using Student’s *t* test (*, *P* < 0.05; **, *P* < 0.01). ‘+’ represents the introduction of exogenous genes or overexpression of endogenous genes, while its number represents the copy number of the respective gene in the yeast strain

Currently, three distinct types of enzymes have been reported to convert retinal into retinol, including alcohol dehydrogenases (ADHs) of the medium-chain dehydrogenase (MDR) family, short-chain dehydrogenases (SDRs) and aldo-keto reductase (AKR) family (Hong et al. 2015). The optimal temperature of *H. sapiens* AKR (30ºC) is the same as that of *S. cerevisiae*, and specifically catalyzes retinal reduction, while AKR1B10 from *M. tractuosa* had a much higher *k*_cat_/*K*_m_ value than AKR (45,000 vs. 427 mM^−1^ min^−1^) (Hong et al. 2015). As an aldehyde reductase, ybbO has a broad substrate spectrum covering C6-C18 aldehydes, and was shown to promote retinol formation when overexpressed (Fatma et al. 2016; Rodriguez and Atsumi 2014). When genes encoding these three enzymes were introduced, respectively, into Y03 after codon optimization, the production of retinol increased to different extents. As the retinol/retinal ratio is determined by the relative activities of the enzymes catalyzing retinal formation and conversion, the higher retinol/retinal ratio suggested higher activity of the retinal reductase. Introduction of AKR and AKR1B10 led to 17.58% and 19.57% more retinol production, respectively (Fig. 5a). To our surprise, 95.79% of retinal was converted into retinol upon ybbO introduction in *S. cerevisiae* albeit at a lower total retinoids production, whereas ybbO overexpression in *E. coli* only resulted in retinol proportions of 53–88% when different resistance markers were used (Jang et al. 2015). Further conversion of retinol to retinyl acetate by chloramphenicol *O*-acetyltransferase used as a chloramphenicol resistance marker and possibly some unknown inherent promiscuous enzymes may explain the lower retinol proportions in *E. coli*.

### Mining of endogenous retinal reductase in* S. cerevisiae*

There is no inherent metabolic pathway related to retinoids in *S. cerevisiae*, but the coproduction of retinal and retinol upon introduction of Blh suggested the presence of endogenous enzymes with nonspecific activity towards retinal. Based on the BLASTp function of NCBI, sequences of Sps19, YMR226C, Ifa38 and Env9 in *S. cerevisiae* showing homology (25.62%, 41.34%, 31.49%, 29.49%) to ybbO were dug out and overexpressed in Y03. Sps19, Ifa38 and YMR226C were reported as a peroxisome-localized 2,4-dienyl-CoA reductase, a 3-ketoreductase of the microsomal fatty acid elongase and a bicyclic diketone reductase, respectively (Gurvitz et al. 1997; Han et al. 2002; Johanson et al. 2008). *ENV9* encodes a conserved lipid droplet (LD) short-chain dehydrogenase involved in LD morphology (Siddiqah et al. 2017). Env9 was implied to be an ortholog of human SDR retinol dehydrogenase 12 (RDH12) based on phylogenetic studies (Siddiqah et al. 2017), but its activity towards retinal was not examined before. Among these four genes, only when *ENV9* was overexpressed, retinol production was significantly improved, reaching 83.63 mg/L, and the retinol-to-retinal ratio was increased to about 2:1, suggesting the retinal reductase activity of Env9 in *S. cerevisiae* (Fig. 5a). When *ENV9* was co-overexpressed with the *E. coli ybbO*, the resulting strain Y03-342 produced 115.28 mg/L retinol, which was higher than those of strains overexpressing two copies of *ybbO* or *ENV9* (Fig. 5a).

The retinal reduction activity of Env9 was further confirmed using an in vitro assay by using the crude extract of *E. coli* expressing Env9. Meanwhile, the cofactor-dependent type of Env9 was determined by adding exogenous NADH or NADPH in the reaction mixture. The results showed that the recombinant Env9 successfully synthesized retinol with retinal as the substrate in the presence of NADPH, while no retinol could be detected without NADPH or using NADH as the cofactor, suggesting the NADPH dependence of this enzyme (Additional file 1: Figure S3). This observation was in accordance with the enhanced retinol biosynthesis upon tPos5 overexpression. Due to the presence of endogenous enzymes with retinal reduction activity in *E. coli* such as ybbO, a small amount of retinol was also detected in the presence of NADPH for the negative control strain harboring the empty vector pET-30a.

Previous sequence comparison and phylogenetic analysis have suggested that *ybbO* was originated from a eukaryotic retinol dehydrogenase ancestor transferred from the human intestine to the *E. coli* genome via horizontal gene transfer (Baker 1998). However, the origin of Env9’s retinal reduction activity remains mysterious.

Multiple sequence alignment (Additional file 1: Figure S4) implied ybbO, Env9 and RDH12 all belong to ‘classical’ SDRs, with highly conserved glycine residues (T_21_GCSSGIG_28_) in the cofactor-binding domain and conserved tyrosine and lysine residue (Y_159_AASK_163_) in the active site (Kavanagh et al. 2008; Siddiqah et al. 2017; Takahashi et al. 2009). The C-terminal hydrophobic domain (aa241–265) of Env9, which was presumed to be a lipid droplets (LDs) membrane binding region (Siddiqah et al. 2017), was not found in either ybbO or RDH12, revealing the unique function of Env9 in LDs formation and dynamics besides the shared retinal reduction activity. When *ENV9* was knocked out in Y03, retinol could still be detected in significant amounts, suggesting the presence of other retinal reductases in *S. cerevisiae* (Additional file 1: Figure S5a). Similarly, deletion of *ybbO* in *E. coli* did not completely block retinol formation (Jang et al. 2015). The endogenous genes in *S. cerevisiae* homologous to *AKR* and *AKR1B10* were also tentatively overexpressed in Y03, but none showed obvious retinol formation activity (Additional file 1: Figure S5b).

### Combinatorial pathway engineering and antioxidant addition

To create a yeast strain with both strengthened precursor and cofactor supply, *CrtE03M* and *tPOS5* were co-overexpressed in Y03 strain, generating Y03-252, which produced a total of 264.03 mg/L retinoids, including 42.66 mg/L retinol and 221.37 mg/L retinal. To increase the retinol proportion in the product, the endogenous enzyme with retinal reductase activity Env9 was overexpressed in Y03-252, constructing Y03-42, which produced 93.8 mg/L retinol and 55.78 mg/L retinal. Further introduction of the *E. coli ybbO* gene into Y03-42 resulted in strain Y03-43, with the highest retinol titer of 122.03 mg/L (Fig. 5b).

Due to the unstable nature of the retinoids, they tend to be oxidized at conjugated double bonds and the β-ionone ring in the presence of oxygen, and this process is accelerated by light (Crank; and Pardijanto, 1995). In addition, partial oxidation of retinol forms retinal (Fu et al. 2003). Addition of a proper antioxidant in the culture is expected to prevent the loss of both retinal and retinol and meanwhile prevent the oxidation of retinol to retinal. When the antioxidant BHT was added into the dodecane layer of the culture, the production of retinoids in all strains increased significantly and the retinol proportions were obviously elevated (Fig. 5b), suggesting that the antioxidant did play an important role in the stabilization of retinoids, especially in the form of retinol. Strains Y03-42 and Y03-43 with retinol as the major product showed the highest production improvement. In the presence of BHT, as much as 401.65 mg/L of retinol as the sole product was achieved in Y03-43, which was 10 times higher than the retinol production of Y03 without BHT addition. The excellent performance of BHT in promoting retinol biosynthesis implies the importance of stabilizing the highly reactive compounds during their production and suggests addition of BHT in the organic overlay as a viable strategy for boosting production of secreted hydrophobic natural products with antioxidant activities. Considering that there are different upper limits of BHT addition in different products, the BHT content should be examined before application of the fermented retinol.

To test whether the promoting effect of BHT on retinol production is universal among antioxidants, the effects of other hydrophobic antioxidants such as butyl hydroxyanisole (BHA), green tea polyphenols (GTP) and propyl gallate (PG) on retinoids were also examined in strain Y03-252 with a mixture of retinol and retinol as the products and strain Y03-43 with retinol as the main product, respectively. As shown in Additional file 1: Figure S6, BHA severely impaired cell growth while GTP and PG also decreased the biomass of both strains tested. Nevertheless, addition of GTP and PG improved the retinoids yields in strain Y03-252 (59.53 mg/g and 46.22 mg/g vs. 41.47 mg/g) and Y03-43 (30.43 mg/g and 34.94 mg/g vs. 24.98 mg/g). These results implied the universal benefit of retinoids production from antioxidant addition and suggested the necessity of selecting a proper antioxidant with minimal effect on cell growth.

In strain Y03-43, obvious accumulation of carotenoids was still observed, implying the formation of retinal as a rate-limiting step (Additional file 1: Figure S7a). In Blh, Fe^2+^ is coordinated with His-21, His-78, His-188, and His-192, and Fe^2+^ plays an important role in its activity (Kim et al. 2009). Considering that ferrous ions are susceptible to oxidation to trivalent ions in air, a supplement of 1.44 mM Fe^2+^ was added to the culture after 36 h of fermentation to maintain the catalytic activity of Blh. As a result, the retinol titer was further improved by 9.03%, reaching 443.43 mg/L, with a proportion of 98.76% in total retinoids, accompanied with decreased carotenoids accumulation (Additional file 1: Figure S7b).

### High-density fermentation of retinol

**Fig. 6:**
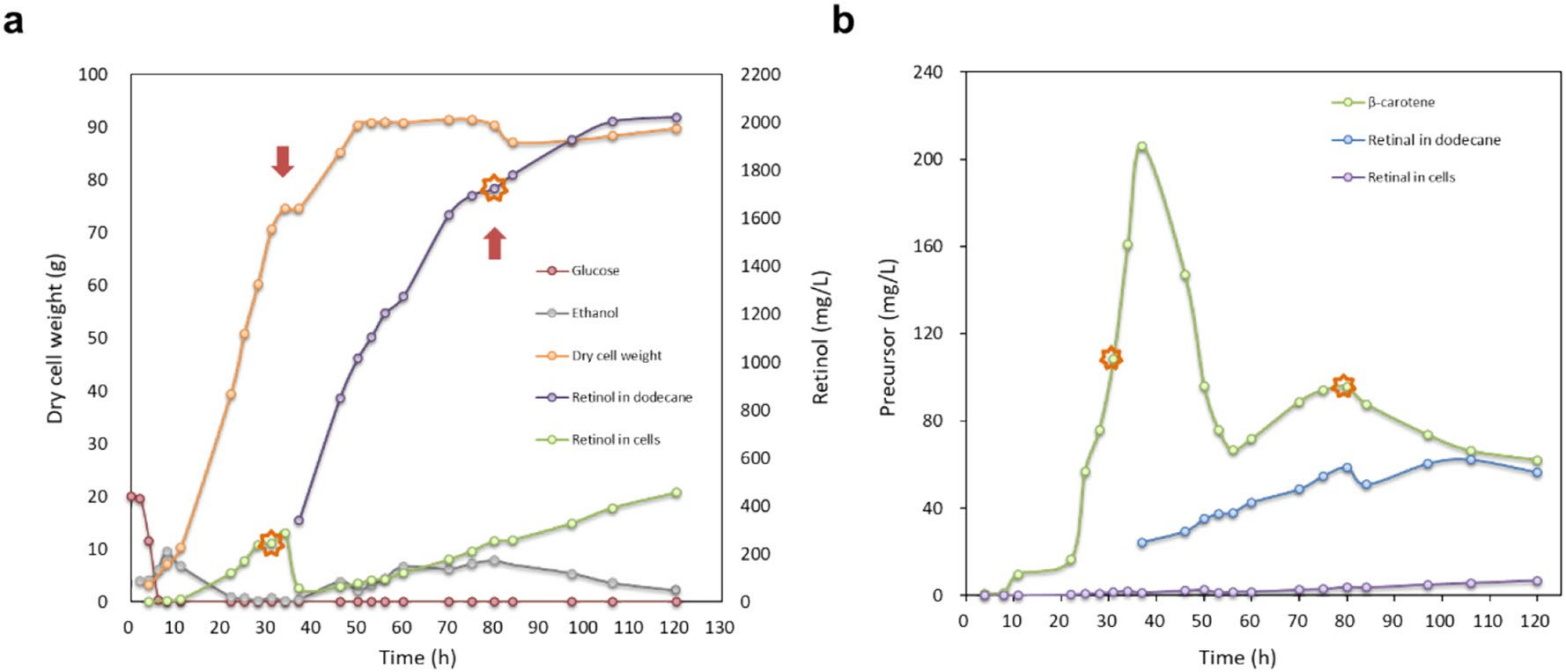
Retinol production of strain Y03-43(+) in fed-batch fermentation. **a** Profile of glucose, ethanol, biomass and retinol accumulation. **b** Accumulation of precursors. The arrows and asterisks indicate the time of adding dodecane and Fe^2+^, respectively

To evaluate the production performance of the engineered strain Y03-43, fed-batch fermentation was conducted in YPD medium with an initial volume of 2 L. At the end of fermentation, the final volume of the aqueous phase and the organic phase was 2.2 L and 180 mL, and the titers of all products were calculated based on the final culture volume. Upon addition of dodecane, retinoids were immediately extracted to the organic phase, leading to obviously accelerated formation of retinoids and sharp consumption of the precursor β-carotene (Fig. 6a). At all stages, the retinol proportion in retinoids was kept above 93.89%. In accordance with the observation in shake-flask cultures, addition of Fe^2+^ at the late stage of fermentation seemed to promote β-carotene conversion and accelerate retinol production (Fig. 6b). Finally, a total retinoids titer of 2542.68 mg/L, consisting of 97.51% retinol (2479.34 mg/L) and 2.49% retinal (63.34 mg/L), was obtained after 120 h of fermentation.

Different from previous studies on retinoids biosynthesis where retinol was produced as one of the components in the retinoids mixture, the yeast strain constructed in this study selectively produced retinol at high titers, which is of great significance for downstream processing and industrial application. However, not all retinol was extracted to the organic phase, with about 18% of retinol trapped in the cells (Fig. 6a), and this problem could not be solved by increasing the volume of extractant. Similar phenomenon was observed in the previous study, where only 83% of the total retinoids was extracted to the dodecane layer even when dodecane was added at a volumetric ratio of 1:1 (Sun et al. 2019). The secretion of hydrophobic small molecules is a complex process involving the cytoplasmic membrane of the yeast cell and rigid cell wall, and the mechanism remains largely unknown (Claus et al. 2019). In the past, the uptake and efflux of small molecules across the plasma membrane were assumed to be passive diffusion (Yang and Hinner 2015). However, more and more studies have revealed the significance of protein-mediated transport in hydrophobic compounds. For instance, fatty acid transport proteins (FATP) and the yeast pleiotropic drug resistance (PDR) transporters play important roles in the transport of different hydrophobic substances (Golin et al. 2003; Zou et al. 2002). If the protein(s) responsible for retinol transport could be identified and properly engineered, the intracellular accumulation problem of retinol in the high-density fermentation might be solved and the retinol production might be further improved.

As compared to the previous study on retinoids biosynthesis using *S. cerevisiae* (Sun et al. 2019), we have obtained far higher retinoids titer in shake-flask cultures (448.98 mg/L vs. 23.55 mg/L or 57.61 mg/L) albeit with a lower carbon source concentration (20 g/L glucose vs. 41 g/L glucose or 43 g/L xylose), demonstrating the efficiency of strengthening precursor and cofactor supply as well as antioxidant addition. However, the final retinoids titer in fed-batch fermentation was lower (2.5 g/L vs. 3.3 g/L), which may largely be caused by the different calculation methods of retinoids titer during the fed-batch fermentation. Nevertheless, combining the pathway optimization strategies validated in the present study and the xylose utilization capability reported in the previous study may lead to further improved biomass and retinol production, considering the negative impact of Crabtree effect on glucose fermentation (Sun et al. 2019).

## Conclusion

For selective and efficient production of the highly valuable vitamin A component retinol, a retinoids-producing yeast was constructed based on a previously constructed β-carotene high-producing yeast strain, followed by systematic engineering to enhance the precursor and NADPH supply, and overexpression of exogenous and endogenous enzymes with retinal reductase activity. Env9 was identified as an endogenous retinal reductase by in vivo and in vitro experiments. Addition of the antioxidant BHT was found effective in stabilizing retinol during bi-phasic fermentation. Finally, the highest ever reported retinol production of 2479.34 mg/L was achieved. This work would lay a foundation for biotechnological production of retinol.

## Materials and methods

### Strains, culture media, reagents

*E. coli* BL21 was used for gene cloning and plasmid amplification. All *S. cerevisiae* strains used in this study are listed in Table 2. Yeast strains were cultivated in YPD medium (1% yeast extract, 2% peptone, and 2% D-glucose). And the yeast cells carrying plasmids with the *URA3* marker were selected and cultivated in SD medium without uracil. SD complete medium with 1 mg/mL of 5-fluoroorotic acid was used for counter-selection of recombinants containing the *URA3* marker.

**Table 2:** *S. cerevisiae* strains used and constructed in this study

Strain name	Parent strain	Genotype/description	Source
Ycarot-02	BY4741	*Δho*::T_*TPS1*_-*tHMG1*-P_*GAL7*_-P_*GAL2*_-*CrtYB*-T_*PGK1*_-T_*CYC1-*_*CrtI*-P_*GAL1*_-P_*GAL10*_-*CrtE03M*-T_*ADH1*_, *Δgal1-7*::T_*ADH1*_-*CrtYB*-P_*GAL10*_-P_*GAL1*_-*CrtI*-T_*CYC1*_, *Δgal80*::*LEU*	Brachmann et al. (2016), Zhou et al. (2017)
Y03	Ycarot-02	*Δlpp1*::P_*GAL1*_-*BLH*-T_*CYC1*_	This study
Y03-11	Y03	*Δdpp1*::P_*GAL7*_-*CrtE03M*-T_*TPS1*_	This study
Y03-12	Y03	P_*ERG9*_::P_*HXT1*_	This study
Y03-13	Y03	*Δrox1*	This study
Y03-14	Y03	*Δmot3*	This study
Y03-15	Y03	*Δypl062w*	This study
Y03-111	Y03-11	P_*ERG9*_::P_*HXT1*_	This study
Y03-112	Y03-11	*Δrox1*	This study
Y03-113	Y03-11	*Δmot3*	This study
Y03-21	Y03	*Δdpp1*::P_*GAL2*_-*YMR315W*-T_*PGK1*_	This study
Y03-211	Y03	*Δdpp1*::T_*TPS1*_-*YMR315W*-P_*GAL7*_-P_*GAL2*_-*tPOS5*-T_*PGK1*_	This study
Y03-22	Y03	*Δdpp1*::P_*GAL2*_-*ZWF1*-T_*PGK1*_	This study
Y03-221	Y03	*Δdpp1*::T_*TPS1*_-*ZWF1*-P_*GAL7*_-P_*GAL2*_-*tPOS5*-T_*PGK1*_	This study
Y03-23	Y03	*Δdpp1*::P_*GAL2*_-*STB5*-T_*PGK1*_	This study
Y03-24	Y03	*Δdpp1*::P_*GAL2*_-*POS5*-T_*PGK1*_	This study
Y03-25	Y03	*Δdpp1*::P_*GAL2*_-*tPOS5*-T_*PGK1*_	This study
Y03-251	Y03	*Δmot3*::P_*GAL10*_-*tPOS5*-T_*ADH1*_	This study
Y03-252	Y03	*Δmot3*::T_*ADH1*_-*tPOS5*-P_*GAL10*_-P_*GAL1*_-*CrtE03M*-T_*CYC1*_	This study
Y03-31	Y03	*Δdpp1*::P_*GAL7*_-*AKR*-T_*TPS1*_	This study
Y03-32	Y03	*Δ*d*pp1*::P_*GAL7*_-*AKR1B10*-T_*TPS1*_	This study
Y03-33	Y03	*Δdpp1*::P_*GAL7*_-*ybbO*-T_*TPS1*_	This study
Y03-331	Y03	*Δdpp1*:: T_*TPS1*_-*ybbO*-P_*GAL2*_-P_*GAL7*_-*ybbO*-T_*TPS1*_	This study
Y03-34	Y03	*Δdpp1*::P_*GAL7*_-*ENV9*-T_*TPS1*_	This study
Y03-341	Y03	*Δdpp1*:: T_*TPS1*_-*ENV9*-P_*GAL2*_-P_*GAL7*_-*ENV9*-T_*TPS1*_	This study
Y03-342	Y03	*Δdpp1*:: T_*TPS1*_-*ybbO*-P_*GAL2*_-P_*GAL7*_-*ENV9*-T_*TPS1*_	This study
Y03-35	Y03	*Δdpp1*::P_*GAL7*_-*SPS19*-T_*TPS1*_	This study
Y03-36	Y03	*Δdpp1*::P_*GAL7*_-*IFA38*-T_*TPS1*_	This study
Y03-37	Y03	*Δdpp1*::P_*GAL7*_-*YMR226C*-T_*TPS1*_	This study
Y03-38	Y03	*Δenv9*	This study
Y03-41	Y03	*Δmot3*::T_*ADH1*_-*tPOS5*-P_*GAL10*_-P_*GAL1*_-*ENV9*-T_*CYC1*_	This study
Y03-42	Y03-41	*Δdpp1*::P_*GAL7*_-*CrtE03M*-T_*TPS1*_	This study
Y03-43	Y03-41	*Δdpp1*::P_*GAL2*_-*CrtE03M*-T_*PGK1*_-P_*GAL7*_-*ybbO*-T_*TPS1*_	This study
Y03-43( +)	Y03-43	*met15*::*MET15*, *his3*::*HIS3*, *ura3*::*URA3*	This study

The media components were purchased from Angel yeast Co., Ltd (Yichang, China). The antibiotics and amino acids were purchased from Sangon Biotech (Shanghai, China). All restriction enzymes, T4 DNA ligase and PrimeSTAR HS DNA polymerase were purchased from Takara (Dalian, China). DNA sequencing was performed by Tsing Biotech Co., Ltd. (Hangzhou, China). NADH and NADPH were purchased from Sangon Biotech (Shanghai, China) and Ruixin Biotechnology Co. Ltd. (Suzhou, China), respectively. And the kits for the detection of NADH and NADPH were purchased from Comin Biotechnology Co. Ltd. (Suzhou, China). The reference compounds of lycopene and retinol were purchased from Solarbio Science & Technology Co., Ltd. (Beijing, China), β-carotene was from XinHeCheng Co., Ltd. (Shaoxing, China), and retinal was from Macklin Biochemical Co., Ltd. (Shanghai, China). Butyl hydroxyanisole (BHA), green tea polyphenols (GTP) and propyl gallate (PG) were purchased from Aladdin biotech Co., Ltd. (Shanghai, China).

### Construction of plasmids and strains

The amino acid sequences of *BLH* (accession number: AAY68319), *AKR* (accession number: ADR23652), and *AKR1B10* (accession number: AAP35440) were codon optimized and synthesized by Generay Biotech Co., Ltd (Shanghai, China). The *ybbO* gene was amplified from *E. coli*, and all yeast genes were amplified from the genomic DNA of *S. cerevisiae* strain Ycarot-02 (Zhou et al. 2017). All these genes were cloned into the multiple cloning sites (MCS) of the previously constructed pUMRI vectors (Xie et al. 2015a) by routine restriction digestion and ligation. The constructed plasmids were transformed into the yeast chromosome by the LiAc/SS carrier DNA/PEG method (Gietz and Schiestl 2007). All primers were synthesized at Generay Biotech Co., Ltd (Shanghai, China). Details of the recombinant plasmids and primers are shown in Additional file 1: Table S1.

### Culture and fermentation conditions

Single colonies were picked into 5 mL of YPD medium and incubated overnight at 30 ºC, 220 rpm. The precultures were then inoculated into shake flasks containing 50 mL YPD medium to an initial OD_600_ of 0.05 and cultured for 84 h. To ensure the Fe^2+^ supply required for the catalytic activity of BCMO, FeSO_4_ solution filtered through 0.22-μm membrane was added to the medium with a final concentration of 1.44 mM. In the bi-phasic fermentation system, a layer of 2.5 ml dodecane (Macklin Biochemical Co., Ltd, Shanghai, China) or dodecane containing 1% (w/v) butylated hydroxytoluene (BHT) (Sigma-Aldrich, St. Louis, MO, USA) was added to the medium for in situ product extraction. In bi-phasic fermentation, the carotenoids and retinoids concentrations were calculated based on the volume of the aqueous phase. Since light would accelerate oxidative degradation of retinoids, shading treatment was used in the process of culture. To determine the dry cell weight, the cells collected from 50 mL of culture were dried at 99 ºC to a constant weight.

### Analysis of carotenoids and retinoids

Carotenoids were extracted and analyzed as previously described (Xie et al. 2015a).The retinoids in the cell pellet were extracted in the same manner as the carotenoids. To determine the extracellular retinoids, the dodecane layer and culture media were harvested by centrifugation. The retinoids in culture media were extracted with equal volume of ethyl acetate and then concentrated by rotary evaporation. The retinoids extracts were diluted with acetone before measurement on the HPLC. For preparation of the retinoids standards, the reference compound was dissolved in methanol and diluted with acetone. Retinoids were quantified on a C18-H column (4.6 × 250 mm, 5 µm, YMC-Pack ODS-AQ, Japan) at 40 °C, and the detection wavelength was set to 352 nm. The mobile phase was 95% methanol and 5% acetonitrile at a flow rate of 0.6 mL/min. All standards or samples of carotenoids and retinoids were freshly prepared and 1% BHT added as an antioxidant during the measurement process.

### Fed-batch fermentation

For fed-batch fermentation, strain Y03-43(+) was constructed by complementing the auxotroph markers in Y03-43. The seed culture was cultivated to a final OD_600_ of 8–12 at 30 °C, 220 rpm, and inoculated (10%, v/v) into a 5 L bioreactor (Shanghai Baoxing Co., Ltd, China) containing 2 L YPD medium supplemented with 20 mL of concentrated trace metal solution. The dissolved oxygen (DO) concentration was kept above 30% of atmospheric oxygen by adjusting the agitation speed from 300 to 550 rpm with a constant air input flow rate of 2.0 vvm, and pH was maintained at 5.0 by automatic addition of ammonium hydroxide. Feeding was conducted using a two-stage strategy, with 500 g/L concentrated glucose solution and 300 g/L concentrated yeast extract solution fed periodically to support cell growth, and 400 g/L ethanol fed at the production stage to provide acetyl-CoA for retinoids biosynthesis. The ethanol concentration was controlled below 5 g/L during the whole process. In the mid-log phase of cell growth, 200 mL dodecane (10%, v/v) together with 1.44 mM FeSO_4_·7H_2_O were added. After 80 h of fermentation, another 30 mL dodecane and 1.44 mM FeSO_4_·7H_2_O were added to make up for the loss of organic solvent and oxidation of Fe^2+^ during the fermentation. The titers of all products were calculated based on the actual culture volume, taking the volume change caused by feeding and sample collection into account.

### Multiple sequence alignment

The multiple sequence alignment was conducted by Fast Fourier Transform (MAFFT) program (Katoh 2002).

### In vitro* enzyme assay of Env9*

To detect the in vitro reducing activity of Env9 on retinal and its coenzyme-dependent phenotype, it was expressed on pET-30a and introduced into *E. coli* BL21. Recombinant *E. coli* strains were cultured and induced by reference to common methods (Ge et al. 2020). The reaction system (1 mL) consisted of 400 μM NADPH or NADH, 120 μL crude enzyme, and 90 mg/L retinal in 100 mM sodium phosphate buffer solution (pH 7). The reaction was conducted at 30 °C for 120 min, and the enzyme activity was assayed by measuring the rate of reduction in NADPH or NADH absorbance values as well as the amounts of retinol formed.

### Supplementary Information

** Additional file 1: Figure S1.** Effect of isopropyl tetradecanoate as an extractant on the β-carotene formation in strain Y03. Additional Figure S1. Effect of isopropyl tetradecanoate as an extractant on the β-carotene formation in strain Y03.** Figure S2.** Effect of increasing *BLH* and *CrtYB* copy number on retinoids production.** Figure S3. ***In vitro* activity of Env9 towards retinoids** Figure S4.** Protein sequence alignment of ybbO, Env9 and RDH12.** Figure S5. **Effects of *ENV9* knockout (**a**) and overexpression of genes homologous to *AKR* and *AKR1B10* (**b**) on retinol synthesis in Y03.** Figure S6. **Effects of different antioxidants on retinoids production by strains Y03-252 and Y03-43.** Figure S7. **Effects of additional Fe^2+^ supplementation after 36 hours of incubation on carotenoids accumulation (**a**) and retinoids production (**b**) of Y03-43.** Table S1. **Plasmids and primers used in this study

## Data Availability

All data supporting this article’s conclusion are available.

## Abbreviations

VAD: Vitamin A deficiency
GGPP: Geranylgeranyl pyrophosphate
MVA: Mevalonic acid
BHT: Butylated hydroxytoluene
BCDO: β-Carotene 15,15′-dioxygenase
BCMO: β-Carotene 15,15′-monooxygenase
ACO: Apo-carotenoid 15,15′-oxygenase
ALDH: Aldehyde dehydrogenase
SDR: Short-chain dehydrogenase/reductase
AKR: Aldo-keto reductase
ADH: Alcohol dehydrogenase
FPP: Farnesyl pyrophosphate
HMG-CoA: 3-Hydroxy-3-methyl-glutaryl-CoA
IPP: Isopentenyl pyrophosphate
DMAPP: Dimethylallyl pyrophosphate
MDR: Medium-chain dehydrogenase
LDs: Lipid droplets
DCW: Dry cell weight
BHA: Butyl hydroxyanisole
GTP: Green tea polyphenols
PG: Propyl gallate
