# Supplementary material for: Selective biosynthesis of retinol in S. cerevisiae
Source: Bioresour Bioprocess. 2022 Mar 12;9(1):22. doi: 10.1186/s40643-022-00512-8 (PMC10991881; doi:10.1186/s40643-022-00512-8)
Supplement: Supplementary file 1 — Additional file 1: Figure S1. Effect of isopropyl tetradecanoate as an extractant on the β-carotene formation in strain Y03. Additional Figure S1. Effect of isopropyl tetradecanoate as an extractant on the β-carotene formation in strain Y03. Figure S2. Effect of increasing BLH and CrtYB copy number on retinoids production. Figure S3. In vitro activity of Env9 towards retinoids Figure S4. Protein sequence alignment of ybbO, Env9 and RDH12. Figure S5. Effects of ENV9 knockout (a) and overexpression of genes homologous to AKR and AKR1B10 (b) on retinol synthesis in Y03. Figure S6. Effects of different antioxidants on retinoids production by strains Y03-252 and Y03-43. Figure S7. Effects of additional Fe2+ supplementation after 36 hours of incubation on carotenoids accumulation (a) and retinoids production (b) of Y03-43. Table S1. Plasmids and primers used in this study [file 40643_2022_512_MOESM1_ESM.docx]

**Additional materials**

**Selective biosynthesis of retinol in *S. cerevisiae***

Qiongyue Hu^a^, Tanglei Zhang^a^, Hongwei Yu^a *^, Lidan Ye^a, b*^

^a^ Institute of Bioengineering, College of Chemical and Biological Engineering, Zhejiang University, Hangzhou 310027, China

^b^ Hangzhou Global Scientific and Technological Innovation Center, Zhejiang University, Hangzhou 311200, China

**
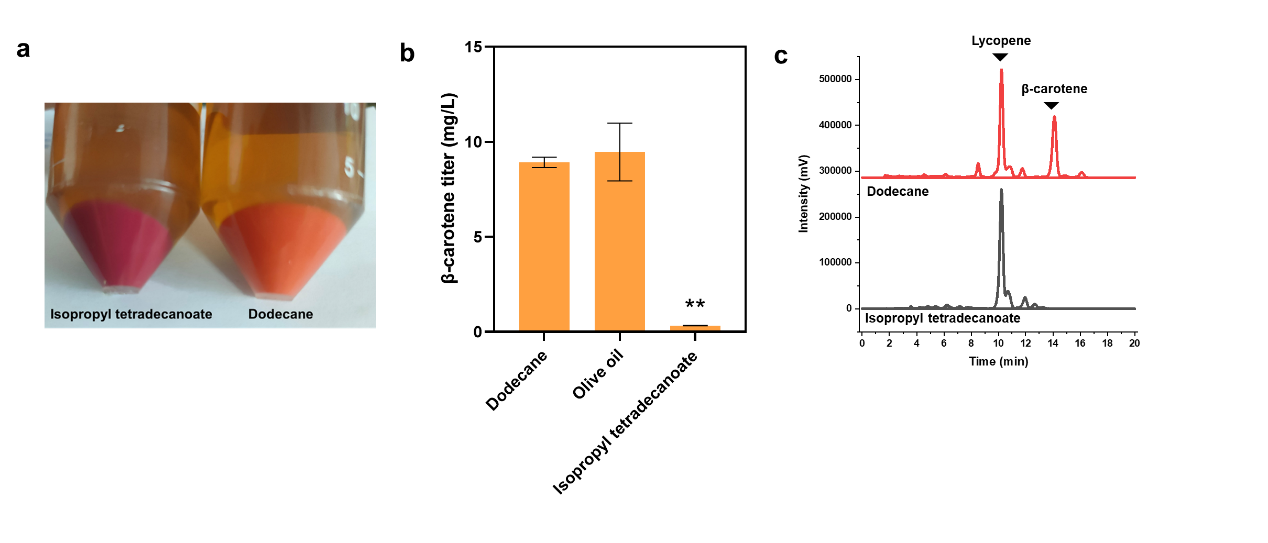
**

**Additional Figure S1. Effect of isopropyl tetradecanoate as an extractant on the β-carotene formation in strain Y03.** (a) Color change of strain Y03 in bi-phase fermentation systems with different organic solvents. (b) β-carotene accumulation of strain Y03 in bi-phase fermentation systems with different organic solvents. (c) HPLC results of strain Y03 cultured with dodecane and isopropyl tetradecanoate as the organic overlay respectively. Statistical significance of the different retinol levels in comparison with the dodecane culture was evaluated using Student’s *t* test (*, *P* < 0.05; **, *P* < 0.01).

**Additional Figure S2 Effect of increasing *BLH* and *CrtYB* copy number on retinoids production.**

**
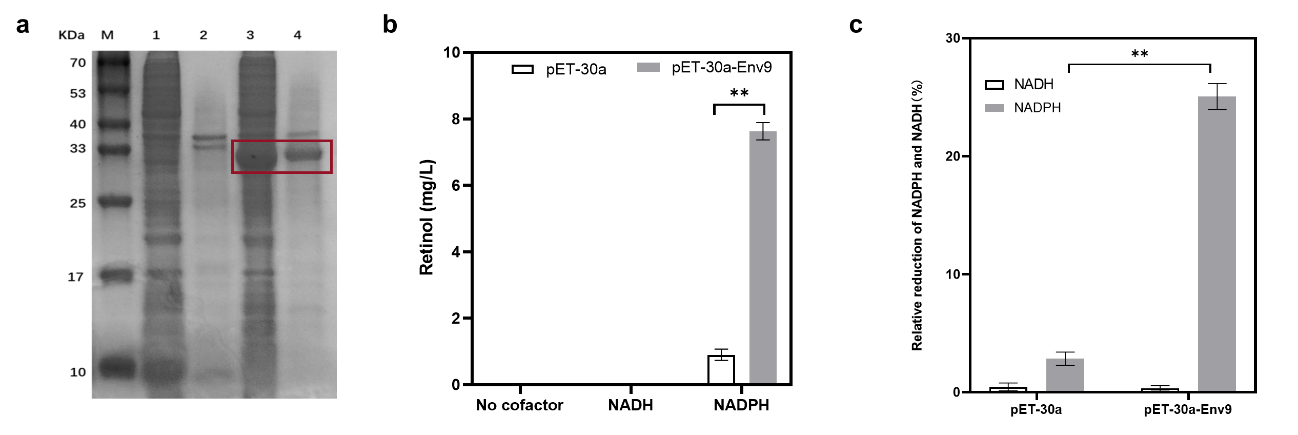
**

**Additional Figure S3** ***In vitro* activity of Env9 towards retinoids.** (a) SDS-PAGE of Env9. M, protein marker. Lanes 1, 2, 3, 4 represent the supernatant and precipitate after disruption of *E. coli* cells harboring pET-30a and pET30a-Env9, respectively. (b) Retinol production by cell extracts of *E. coli* BL21(DE3) expressing pET30a or pET30a-Env9 in the presence of NADH or NADPH. (c) Relative reduction of NADH or NADPH at the end of reaction with the cell extract of *E. coli* harboring pET30a-Env9. The reaction system (1 mL) consisted of 400 μM NADPH or NADH, 120 μL crude enzyme, and 90 mg/L retinal in 100 mM sodium phosphate buffer solution (pH 7.0). The reaction was conducted at 30 ºC for 120 min. Statistical significance was evaluated using Student’s *t* test (*, *P* < 0.05; **, *P* < 0.01).


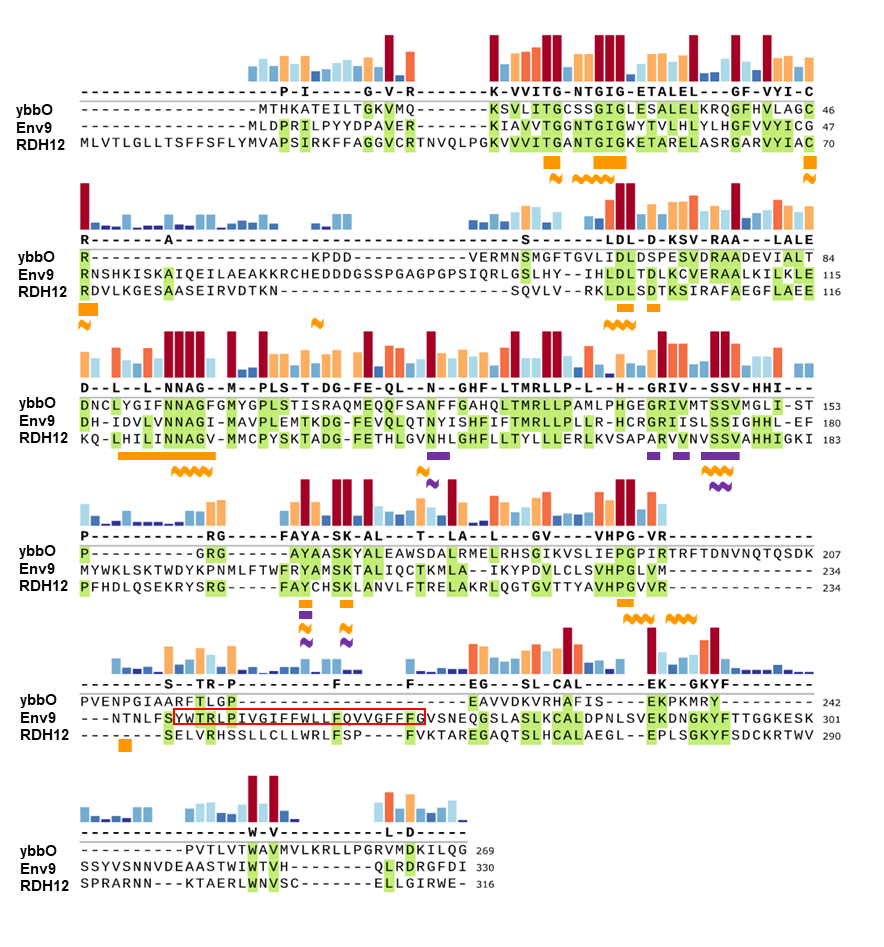


**Additional Figure S4** **Protein sequence alignment of ybbO, Env9 and RDH12.** The similarity of the aligned residues is indicated by colored bars (with warm colors corresponding to high similarity and cool colors to low similarity, and the column height representing the degree of similarity). The thick orange lines below the sequences represent the reported coenzyme binding sites of Env9, and the thick purple lines represent its active center, and the predicted lipid droplets binding region (aa241-246) is shown in red box ([Siddiqah et al., 2017](#_ENREF_2)). As for ybbO, the orange thick wavy lines represent the predicted coenzyme binding sites, and the purple thick wavy lines represent the active site residues.


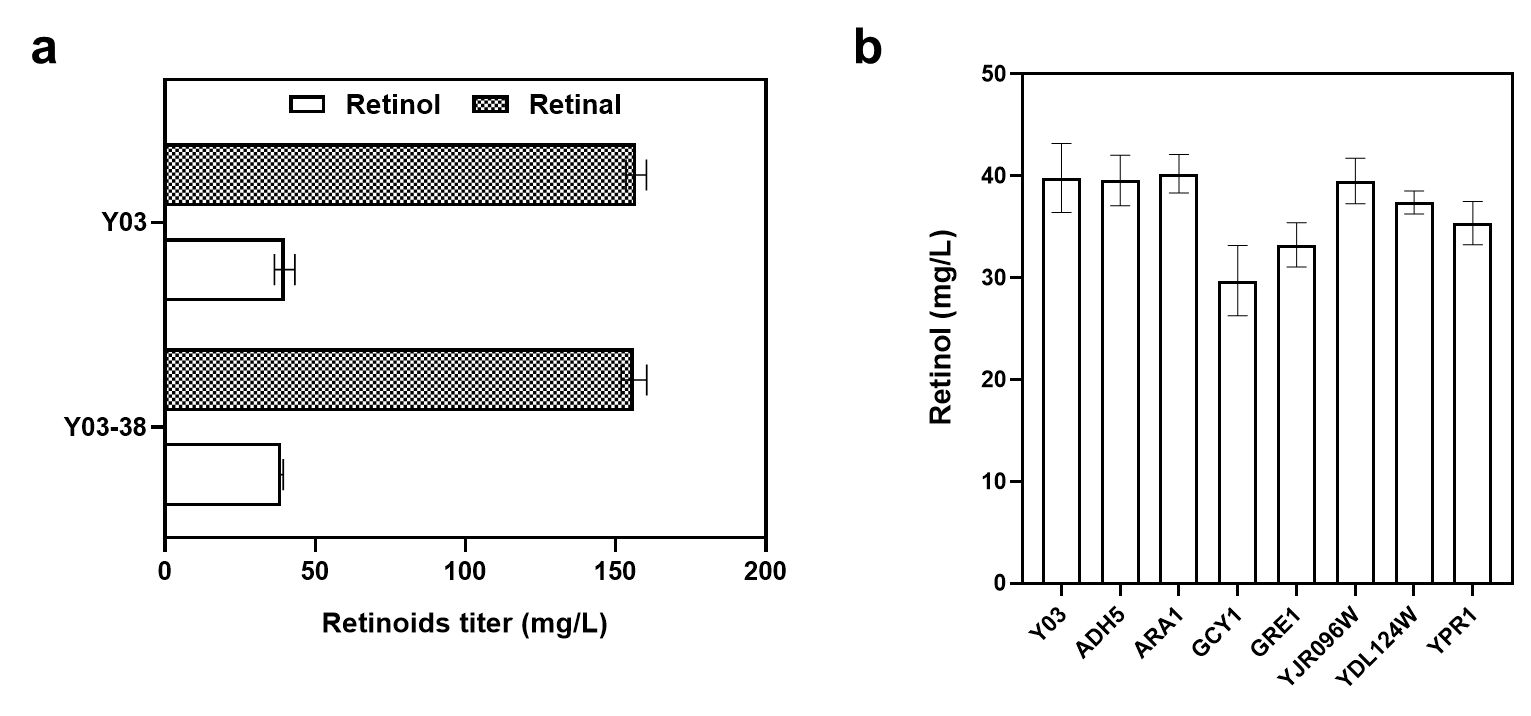


**Additional Figure S5** **Effects of *ENV9* knockout (a) and overexpression of genes homologous to *AKR* and *AKR1B10* (b) on retinol synthesis in Y03.**

**
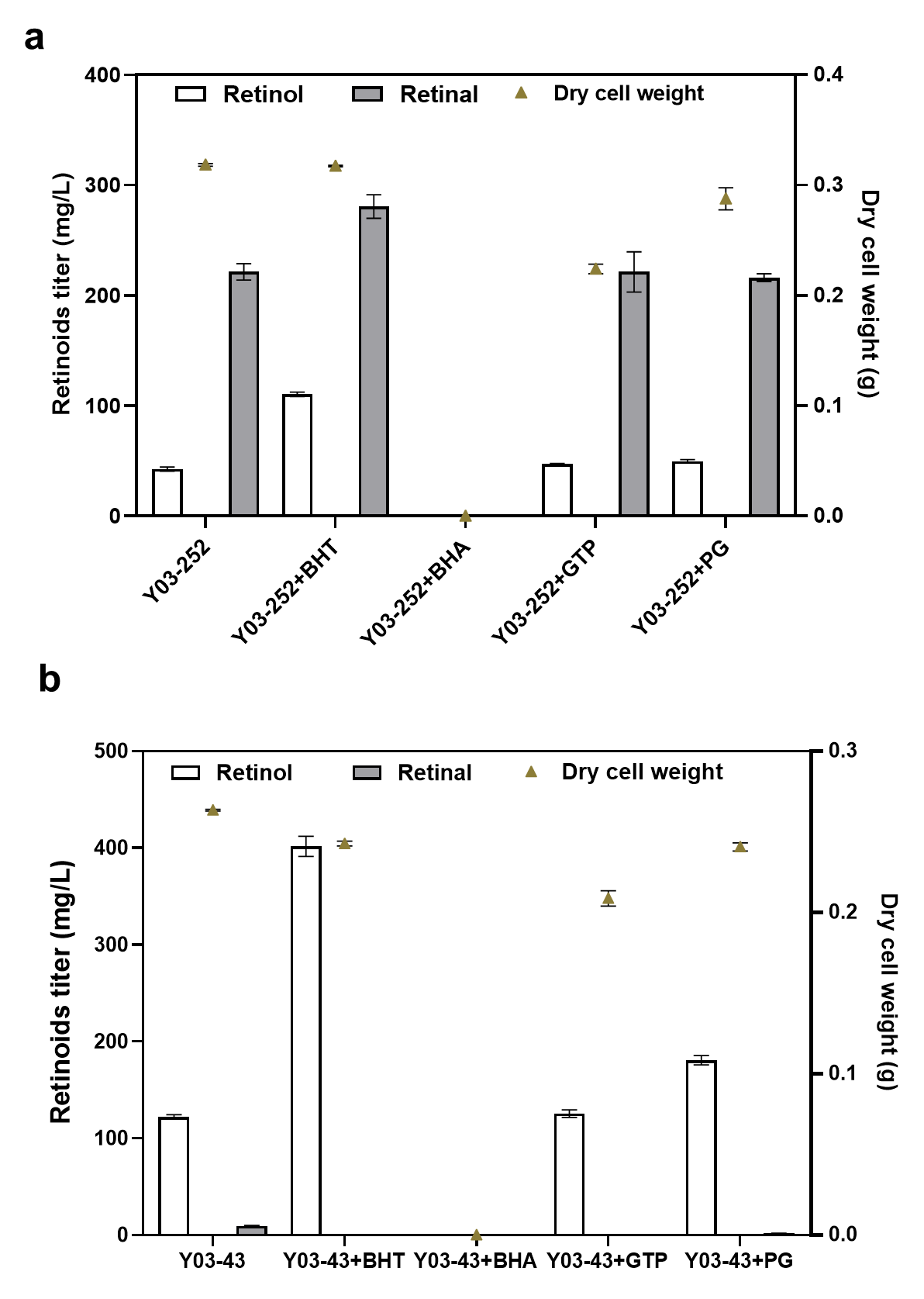
**

**Additional Figure S6** **Effects of different antioxidants on retinoids production by strains Y03-252 and Y03-43.** The antioxidants and their concentrations were 1% BHT, 0.1% BHA, 0.1% GTP and 0.01% PG, respectively.

**
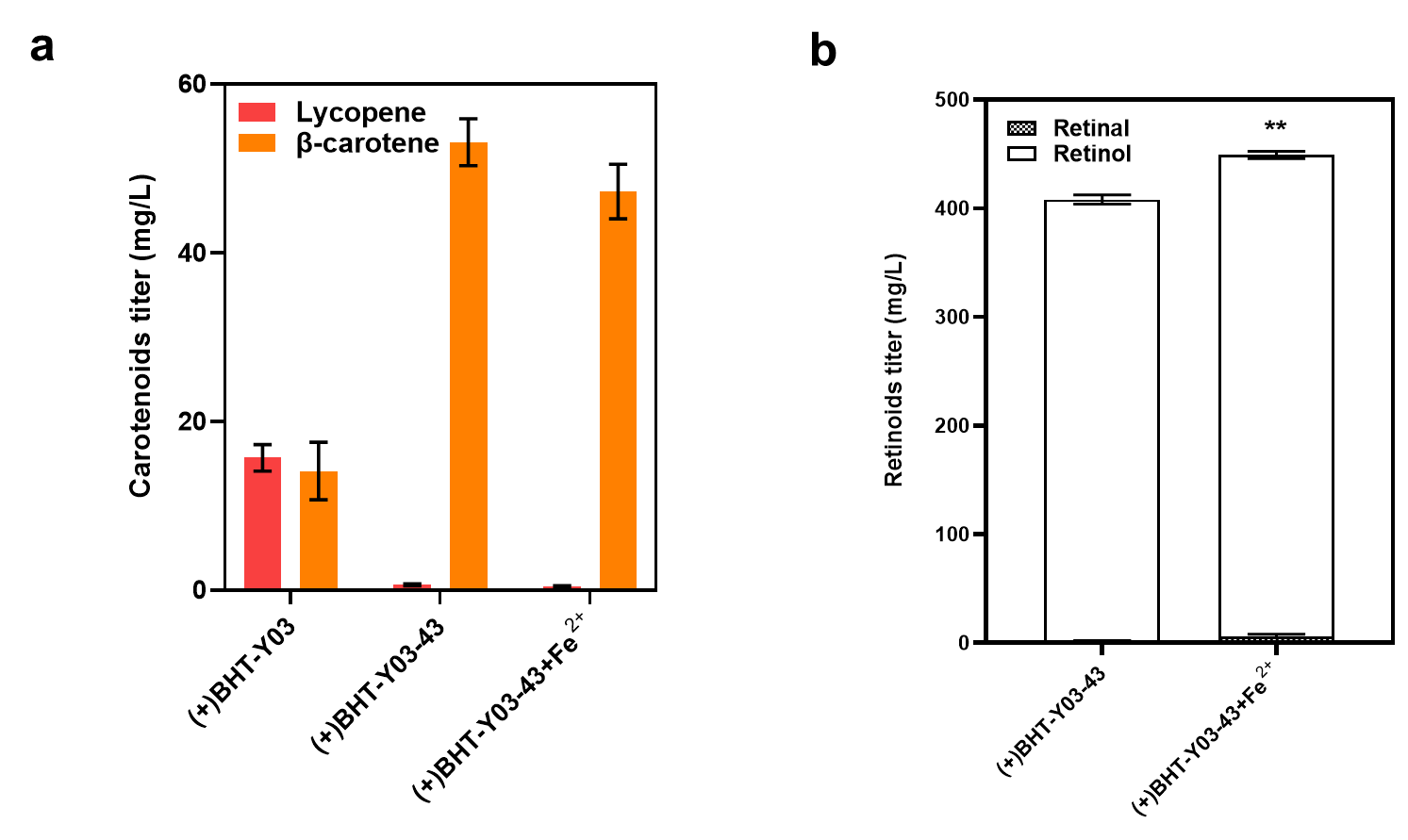
**

**Additional Figure** **S7 Effects of additional Fe^2+^ supplementation after 36 hours of incubation on carotenoids accumulation (a) and retinoids production (b) of Y03-43.** Statistical significance was evaluated using Student’s *t* test (*, *P* < 0.05; **, *P* < 0.01).

**Additional Table S1. Plasmids and primers used in this study**

| **Plasmid name** | **Genotype/ Description** | **Reference** |
| --- | --- | --- |
| pUMRI-LPP1 | *loxp*-*KanMX*-*URA3*-*pbr322ori*-*loxp*,  T*_ADH1_*-*MCS1*-P*_GAL10_*-P*_GAL1_*-*MCS2*-T*_CYC1_*,  *LPP1* homologous arm | ([Shen et al., 2020](#_ENREF_1)) |
| pUMRI-DPP1 | *loxp*-*KanMX*-*URA3*-*pbr322ori*-*loxp*,  T*_PGK1_*-*MCS1*-P*_GAL2_*-P*_GAL7_*-*MCS2*-T*_TPS1_*,  *DPP1* homologous arm | This study |
| pUMRI-ROX1 | *loxp*-*KanMX*-*URA3*-*pbr322ori*-*loxp*,  T*_ADH1_*-*MCS1*-P*_GAL10_*-P*_GAL1_*-*MCS2*-T*_CYC1_*,  *ROX1* homologous arm | This study |
| pUMRI-MOT3 | *loxp*-*KanMX*-*URA3*-*pbr322ori*-*loxp*,  T*_ADH1_*-*MCS1*-P*_GAL10_*-P*_GAL1_*-*MCS2*-T*_CYC1_*,  *MOT3* homologous arm | This study |
| pUMRI-YPL062W | *loxp*-*KanMX*-*URA3*-*pbr322ori*-*loxp*,  T*_ADH1_*-*MCS1*-P*_GAL10_*-P*_GAL1_*-*MCS2*-T*_CYC1_*,  *YPL062W* homologous arm | This study |
| pUMRI-ENV9 | *loxp*-*KanMX*-*URA3*-*pbr322ori*-*loxp*,  T*_ADH1_*-*MCS1*-P*_GAL10_*-P*_GAL1_*-*MCS2*-T*_CYC1_*,  *ENV9* homologous arm | This study |
| pUMRI-ERG9 | *loxp*-*KanMX*-*URA3*-*pbr322ori*-*loxp*,  P*_HXT1_*-P*_TEF1_*-*MCS2*-T*_CYC1_*,  *ERG9* homologous arm | This study |
| pUMRI-LPP1-*BLH* | *loxp*-*KanMX*-*URA3*-*pbr322ori*-*loxp*,  T*_ADH1_*-*MCS1*-P*_GAL10_*-P*_GAL1_*-*BLH*-T*_CYC1_*,  *LPP1* homologous arm | This study |
| pUMRI-DPP1-*CrtE03M* | *loxp*-*KanMX*-*URA3*-*pbr322ori*-*loxp*,  T*_PGK1_*-*MCS1*-P*_GAL2_*-P*_GAL7_*-*CrtE03M*-T*_TPS1_*,  *DPP1* homologous arm | This study |
| pUMRI-DPP1-*YMR315W* | *loxp*-*KanMX*-*URA3*-*pbr322ori*-*loxp*,  T*_PGK1_*-*YMR315W*-P*_GAL2_*-P*_GAL7_*-*MCS2*-T*_TPS1_*,  *DPP1* homologous arm | This study |
| pUMRI-DPP1-*YMR315W-tPOS5* | *loxp-KanMX-URA3-pbr322ori-loxp,*  *TPGK1-tPOS5-PGAL2-PGAL7-YMR315W-TTPS1,*  *DPP1 homologous arm* | This study |
| pUMRI-DPP1-*POS5* | *loxp*-*KanMX*-*URA3*-*pbr322ori*-*loxp*,  T*_PGK1_*-*POS5*-P*_GAL2_*-P*_GAL7_*-*MCS2*-T*_TPS1_*,  *DPP1* homologous arm | This study |
| pUMRI-DPP1-*tPOS5* | *loxp*-*KanMX*-*URA3*-*pbr322ori*-*loxp*,  T*_PGK1_*-*tPOS5*-P*_GAL2_*-P*_GAL7_*-*MCS2*-T*_TPS1_*,  *DPP1* homologous arm | This study |
| pUMRI-DPP1-*ZWF1* | *loxp*-*KanMX*-*URA3*-*pbr322ori*-*loxp*,  T*_PGK1_*-*ZWF1-*P*_GAL2_*-P*_GAL7_*-*MCS2*-T*_TPS1_*,  *DPP1* homologous arm | This study |
| pUMRI-DPP1-*ZWF1-tPOS5* | *loxp*-*KanMX*-*URA3*-*pbr322ori*-*loxp*,  T*_PGK1_*-*tPOS5-*P*_GAL2_*-P*_GAL7_-ZWF1*-T*_TPS1_*,  *DPP1* homologous arm | This study |
| pUMRI-DPP1-*STB5* | *loxp*-*KanMX*-*URA3*-*pbr322ori*-*loxp*,  T*_PGK1_-STB5*-P*_GAL2_*-P*_GAL7_*-*MCS2*-T*_TPS1_*,  *DPP1* homologous arm | This study |
| pUMRI-DPP1-*AKR* | *loxp*-*KanMX*-*URA3*-*pbr322ori*-*loxp*,  T*_PGK1_*-*MCS1*-P*_GAL2_*-P*_GAL7_*-*AKR-*T*_TPS1_*, *DPP1* homologous arm | This study |
| pUMRI-DPP1-*AKR1B10* | *loxp*-*KanMX*-*URA3*-*pbr322ori*-*loxp*,  T*_PGK1_*-*MCS1*-P*_GAL2_*-P*_GAL7_*-*AKR1B10*-T*_TPS1_*,  *DPP1* homologous arm | This study |
| pUMRI-DPP1-*ybbO* | *loxp*-*KanMX*-*URA3*-*pbr322ori*-*loxp*,  T*_PGK1_*-*MCS1*-P*_GAL2_*-P*_GAL7_*- *ybbO* -T*_TPS1_*,  *DPP1* homologous arm | This study |
| pUMRI-DPP1-*ybbO*-*ybbO* | *loxp*-*KanMX*-*URA3*-*pbr322ori*-*loxp*,  T*_PGK1_*-*ybbO*-P*_GAL2_*-P*_GAL7_*- *ybbO* -T*_TPS1_*,  *DPP1* homologous arm | This study |
| pUMRI-DPP1-*SPS19* | *loxp*-*KanMX*-*URA3*-*pbr322ori*-*loxp*,  T*_PGK1_*-*MCS1*-P*_GAL2_*-P*_GAL7_*-*SPS19*-T*_TPS1_*,  *DPP1* homologous arm | This study |
| pUMRI-DPP1-*YMR226C* | *loxp*-*KanMX*-*URA3*-*pbr322ori*-*loxp*,  T*_PGK1_*-*MCS1*-P*_GAL2_*-P*_GAL7_*-*YMR226C*-T*_TPS1_*,  *DPP1* homologous arm | This study |
| pUMRI-DPP1-*IFA38* | *loxp*-*KanMX*-*URA3*-*pbr322ori*-*loxp*,  T*_PGK1_*-*MCS1*-P*_GAL2_*-P*_GAL7_*-*IFA38*-T*_TPS1_*,  *DPP1* homologous arm | This study |
| pUMRI-DPP1-*ENV9* | *loxp*-*KanMX*-*URA3*-*pbr322ori*-*loxp*,  T*_PGK1_*-*MCS1*-P*_GAL2_*-P*_GAL7_*-*ENV9*-T*_TPS1_*,  *DPP1* homologous arm | This study |
| pUMRI-DPP1-*ENV9-ENV9* | *loxp*-*KanMX*-*URA3*-*pbr322ori*-*loxp*,  T*_PGK1_*-*MCS1*-P*_GAL2_*-P*_GAL7_*-*ENV9*-T*_TPS1_*,  *DPP1* homologous arm | This study |
| pUMRI-DPP1-*ENV9-ybbO* | *loxp*-*KanMX*-*URA3*-*pbr322ori*-*loxp*,  T*_PGK1_*-*ybbO*-P*_GAL2_*-P*_GAL7_*-*ENV9*-T*_TPS1_*,  *DPP1* homologous arm | This study |
| pUMRI-MOT3-*tPOS5* | *loxp*-*KanMX*-*URA3*-*pbr322ori*-*loxp*,  T*_ADH1_*-*tPOS5*-P*_GAL10_*-P*_GAL1_*-*MCS2*-T*_CYC1_*,  *MOT3* homologous arm | This study |
|  |  |  |
| pUMRI-MOT3-*tPOS5*-*CrtE03M* | *loxp*-*KanMX*-*URA3*-*pbr322ori*-*loxp*,  T*_ADH1_*-*tPOS5*-P*_GAL10_*-P*_GAL1_*-*CrtE03M*-T*_CYC1_*,  *MOT3* homologous arm | This study |
| pUMRI-MOT3-*tPOS5-ENV9* | *loxp*-*KanMX*-*URA3*-*pbr322ori*-*loxp*,  T*_ADH1_*-*tPOS5*-P*_GAL10_*-P*_GAL1_*-*ENV9*-T*_CYC1_*,  *MOT3* homologous arm | This study |
| pUMRI-DPP1-*ybbO-CrtE03M* | *loxp*-*KanMX*-*URA3*-*pbr322ori*-*loxp*,  T*_PGK1_*-*CrtE03M*-P*_GAL2_*-P_GAL7_-*ybbO*-T*_TPS1_*,  *DPP1* homologous arm | This study |
| pET-30a | Kn^R^, pBR322 ori, T7-promoter | Laboratory stock |
| pET-30a-Env9 | Kn^R^, P*_T7_*-*ENV9*-T*_T7_* | This study |

| **Primer name** | **Sequence (5’-3’)** | **Description** |
| --- | --- | --- |
| BLH-BamHI-F | CGCGGATCCATGGGTTTAATGTTAATTGATTGG | For amplification of genes |
| BLH-XohI-R | CCGCTCGAGTTAATTTTTAATTTTAATCCTTGAAGAATGTGGT |  |
| crtE03M-BamHI-F | AGGGGATCCATGGATTACGCGAACATCCTCAC |  |
| crtE03M-kpnI-R | CGTGGTACCTCACAGAGGGATATCGGCTAGCTT |  |
| crtE03M-EcoRI-F | CCGGAATTCATGGATTACGCGAACATCCTCACAGCAATTCCAC |  |
| crtE03M-SacI-R | CGCGAGCTCTCACAGAGGGATATCGGCTAGCTTTTTCAGGAT |  |
| POS5-NotI-F | ATTTGCGGCCGCATGTTTGTCAGGGTTAAATTGAATAAACCAG |  |
| POS5-SacI-R | CGCGAGCTCTTAATCATTATCAGTCTGTCTCTTGGTCAGCCTAA |  |
| STB5-NotI-F | ATTTGCGGCCGCATGGATGGTCCCAATTTTGCACATCAAGGC |  |
| STB5-SacI-R | CGCGAGCTCTCATACAAGTTTATCAACCCAAGAGACGTCAACA |  |
| YMR315W-NotI-F | ATTTGCGGCCGCATGTCCCCATTGAACGTCGGTATAGTTGGTA |  |
| YMR315W-SacI-R | CGCGAGCTCTCATGGCTGCTCAATTTTAACGTAGTCACCATTC |  |
| YMR315W-BamHI-F | CGCGGATCCATGTCCCCATTGAACGTCGGTATAGTTGGTA |  |
| YMR315W-XohI-R | CCGCTCGAGTCATGGCTGCTCAATTTTAACGTAGTCACCATTC |  |
| ZWF1-NotI-F | ATTTGCGGCCGCATGAGTGAAGGCCCCGTCAAATTCGAAAAA |  |
| ZWF1-SacI-R | CGCGAGCTCCTAATTATCCTTCGTATCTTCTGGCTTAGTCACGG |  |
| ZWF1-BamHI-F | CGCGGATCCATGAGTGAAGGCCCCGTCAAATTCGAAAAAAAT |  |
| ZWF1-XohI-R | CCGCTCGAGCTAATTATCCTTCGTATCTTCTGGCTTAGTCACGGG |  |
| tPOS5-NotI-F | ATTGCGGCCGCATGAGTACGTTGGATTCACATTCCCTAAAGTT |  |
| sthA-NotI-F | ATTGCGGCCGCATGCCACATTCCTACGATTACGATGCC |  |
| sthA-SacI-R | ACCGAGCTCTTAAAACAGGCGGTTTAAACCGTTTAA |  |
| AKR1B10-BamHI-F | CGCGGATCCATGGCAACTTTTGTTGAATTATCTACTAAAGCTA |  |
| AKR1B10-XhoI-R | CCGCTCGAGTTAATATTCTGCATCAAATGGATAATCTTCTAAAT |  |
| AKR-BamHI-F | CGCGGATCCATGAGGAAATTAACTTTTAGGAA |  |
| AKR-XhoI-R | CCGCTCGAGTTATTCACCCCACAAACCA |  |
| ybbO-BamHI-F | CGCGGATCCATGACTCATAAAGCAACGGAGATCCTGACAGGT |  |
| ybbO-XhoI-R | CCGCTCGAGTCACCCCTGCAATATTTTGTCCATCACGCGCCC |  |
| ybbO-EcoRI-F | CGCGAATTCATGACTCATAAAGCAACGGAGATCCTGACAGGTAAAGTTAT |  |
| ybbO-SacI-R | CCGGAGCTCTCACCCCTGCAATATTTTGTCCATCACGCGCCC |  |
| YMR226C-BamHI-F | CGCGGATCCATGTCCCAAGGTAGAAAAGCTGCAGAAAGATTG |  |
| YMR226C-SalI-R | CCGGTCGACTTATCCACGGAAGATATGATGAGGTGACGCTTG |  |
| ENV9-BamHI-F | CGCGGATCCATGTTAGACCCACGAATATTGCCATACTACGACC |  |
| ENV9-XhoI-R | CCGCTCGAGTTATATATCGAAACCACGGTCTCTTAGTTGATGA |  |
| ENV9-EcoRI-F | CGCGAATTCATGTTAGACCCACGAATATTGCCATACTACGACCCG |  |
| ENV9-SacI-R | CGGGAGCTCTTATATATCGAAACCACGGTCTCTTAGTTGATGA |  |
| IFA38-BamHI-F | CGCGGATCCATGACTTTTATGCAACAGCTTCAAGAGGCTGGG |  |
| IFA38-SalI-R | CCGGTCGACCTATTCCTTTTTAACCTGTCTTGCGGCTTTTT |  |
| SPS19-BamHI-F | CGCGGATCCATGAATACAGCAAACACTTTGGACGGCAAATT |  |
| SPS19-SalI-R | CCGGTCGACTTATAATTTAGATGTCATACTCTTTATTAAGGCTT |  |
| ADH5-BamHI-F | CGCGGATCCATGTCCGCCGCTACTGTTGGTAAACCTATT |  |
| ADH5-XhoI-R | CCGCTCGAGCTATTTTATTTCATCAGACTTCAAGACGGTT |  |
| ARA1-BamHI-F | CGCGGATCCATGTCTTCTTCAGTAGCCTCAACCGAAAACATAGTCG |  |
| ARA1-KpnI-R | CGGGGTACCTTAATACTTTAAATTGTCCAAGTTTGGTCCGTTACCAG |  |
| GCY1-BamHI-F | CGCGGATCCATGCCTGCTACTTTACATGATTCTACGAAAATCCTTTC |  |
| GCY1-XhoI-R | CCGCTCGAGTTACTTGAATACTTCGAAAGGAGACCAATTTGGATGT |  |
| YPR1-SalI-F | GCCGTCGACATGCCTGCTACGTTAAAGAATTCTTCTGCTACATTAAA |  |
| YPR1-XhoI-R | CCGCTCGAGTCATTGGAAAATTGGGAAGGATCCCCACTTCATATCA |  |
| GRE3-BamHI-F | CGCGGATCCATGTCTTCACTGGTTACTCTTAATAACGGTCTGAAAAT |  |
| GRE3-XhoI-R | CCGCTCGAGTCAGGCAAAAGTGGGGAATTTACCATCCAACCAGGT |  |
| YJR096W-BamHI-F | CGCGGATCCATGGTTCCTAAGTTTTACAAACTTTCAAACGGCTTCA |  |
| YJR096W-XhoI-R | CCGCTCGAGTTATGGCGCGTCTGTGCATTCCCAATCGGTAGGCTCA |  |
| YDL124W-BamHI-F | CGCGGATCCATGTCATTTCACCAACAGTTCTTTACCTTGAATAATGG |  |
| YDL124W-XhoI-R | CCGCTCGAGTTATACTTTTTGAGCAGCGTAGTTGTATTTACCGTAC |  |
| MOT3HO-UP-F | CGTCTGGATTTACTAAACTTTGTCTCTCTTTTGATGTAAAGTTAA | For verifying plasmids construction and gene integration |
| MOT3HO-DR-R | GTTGTAATCAGTTCCGTACCATACATAAACTATATGTATATA |  |
| ENV9HO-UP-F | GTACATGGGTATATATAAACAAATCATTATGGTGCATGATG |  |
| ENV9HO-DR-R | CATTACGTCAATGCTGACTGAATCTATCAATTGAATAAA |  |
| DPP1HO-UP-F | CTTCATAAAGGGACAACACGGCTTATAGCATT |  |
| DPP1HO-DR-R | GAAATAACAAGCAGGCCCTTGCACGTCAAGATTA |  |
| LPP1-UP-F | TTCGTTGTTTACCTATTTTCCTGCCAATTCA |  |
| LPP1-DR-R | TCCTTCTTATACGCCTCAAGACGACAGA |  |
| ROX1-UP-F | CCAGTTTTAATGTTTCTTCTCATTGCTTTC |  |
| ROX1-DR-2 | ACTTTTATTTGCTGTTATTTTACATATTTTTCAATTGTTC |  |
| HOR2 | ACTACGGCTACACTAGAAGGACAGTATTTG |  |
| GAL1-F | TTATTTCTGGGGTAATTAATCAGCGAAGC |  |
| GAL7-F | GGGCATTATTATGCAGAGCATCAACATGATAAAA |  |
| GAL10-F | TGATTATTAAACTTCTTTGCGTCCATCCAA |  |
| GAL2-F | TTTGGTGTTGTGAATTGCTCTTCATTATGCACCTT |  |
| HXT1-F | GATTTTACGTATATCAACTAGTTGACGATTATG |  |
| qtHMG1-F | TTCAAATTTTTCTTTTTTTTCTGTACAGACGCGTG |  |
| TPS1-F | CCCTCTTTTTCTCTCTCTTTCTTTCTCTCC |  |
| T7-F | TAATACGACTCACTATAGGGGAATTGTGAGCGGATAACAAT |  |
| HIS3-F | CGTTTTAAGAGCTTGGTGAGC | For complementation of auxotroph markers |
| HIS3-R | CGCCTCGTTCAGAATGACA |  |
| URA3-F | GAAGAATTAATTGAGGGCGGATTACTACCGTTG |  |
| URA3-R | AGATACATAATTAGATATATATACGCCAGT |  |
| MET15-F | AAGTTCTCGTCGAATGCTAGGTC |  |
| MET15-R | GGTGTTGACACCTTCTCCGC |  |

**Additional references**

Shen, B., Zhou, P., Jiao, X., et al., 2020. Fermentative production of Vitamin E tocotrienols in *Saccharomyces cerevisiae* under cold-shock-triggered temperature control. Nature Communications. 11**,** 5155-5169.

Siddiqah, I. M., Manandhar, S. P., Cocca, S. M., et al., 2017. Yeast *ENV9* encodes a conserved lipid droplet (LD) short-chain dehydrogenase involved in LD morphology. Current Genetics. 63**,** 1053-1072.
